# The effectiveness and safety of commercial Chinese polyherbal preparation in treating infantile anorexia: a systematic review and Bayesian network meta-analysis

**DOI:** 10.3389/fphar.2026.1775817

**Published:** 2026-06-18

**Authors:** Rongchen Liu, Jiahui Liu, Linrong Li

**Affiliations:** School of Basic Medical Sciences, Shanxi University of Chinese Medicine, Taiyuan, China

**Keywords:** Bayesian network meta-analysis, commercial Chinese polyherbal preparation, effectiveness, infantile anorexia, safety

## Abstract

**Objective:**

Commercial Chinese polyherbal preparations (CCPPs) are increasingly used for infantile anorexia (IA), but comparative effectiveness and safety differences among them remain unclear. This Bayesian network meta-analysis compares various CCPPs for IA to inform clinical practice.

**Methods:**

We systematically searched CNKI, Wanfang, VIP, China Biology Medicine, PubMed, Web of Science, Embase, and Cochrane Library for RCTs on CCPPs for IA up to 9 April 2024. Study quality was assessed using the Cochrane RoB tool. Primary outcomes were overall effective rate, weight change, hemoglobin (Hb) levels, and adverse reactions. This research implemented Bayesian network meta-regression to ascertain the impact of different CCPPs and durations of treatment on the effectiveness and safety to treat IA.

**Results:**

141 RCTs (n = 16,963 patients; 49 CCPPs) were included. For overall effective rate, Huaji Oral Solution (HJKFY), Jianpi Pills + Reference Drug (JPW + RD), and Erkangning (EKN) showed significant superiority vs. control (all RR > 1.54, P < 0.05). Regarding weight change, Erpixing Granules (EPXKL) and Xiaoer Fufang Jineijin Chewable Tablets + RD (XEFFJNJJJP + RD) demonstrated significant benefits (MD = 2.26 and 2.10, P < 0.05 for XEFFJNJJJP + RD). For Hb levels, Jianbaoling Granules (JBLKL), Erbao Granules + RD (EBKL + RD), and Shenqu Xiaoshi Oral Solution + RD (SQXSKFY + RD) showed significant advantages (all MD > 10.72, P < 0.05). CCPPs exhibited a favorable safety profile, with mostly mild adverse reactions.

**Conclusion:**

CCPPs are significantly more effective and safer than control treatments for IA. Different CCPPs excelled on specific outcomes: HJKFY for overall efficacy, EPXKL for weight gain, and JBLKL for Hb improvement. These findings require confirmation via larger, high-quality, multi-center RCTs.

**Systematic Review Registration:**

https://www.crd.york.ac.uk/prospero/, identifier CRD42024535550.

## 1 Introduction

Infantile anorexia (IA) is a non-organic feeding disorder that typically manifests before the age of 3 years, commonly between 9 and 18 months (Özyurt et al., 2018). Based on the Diagnostic Classification of Mental Health and Developmental Disorders of Infancy and Early Childhood (DC: 0–3R, Zero To Three, 2005), IA is defined based on the following three clinical features: (i) Infants persistently refuse to consume sufficient food for at least 1 month and exhibit growth retardation; (ii) Infants do not display signals of hunger and lack interest in food; (c) Food refusal behaviors caused by traumatic events or underlying somatic diseases are excluded (Lucarelli et al., 2017). Furthermore, according to the International Classification of Diseases, 11th Revision (ICD-11), such non-organic feeding disorder are classified under the category of functional feeding disorders in infants and toddlers (World Health Organization, 2022). However, according to the Diagnostic and Statistical Manual of Mental Disorders, Fifth Edition (DSM-5), such clinical presentations are classified as avoidant/restrictive food intake disorder (ARFID), which serves as an important subtype of feeding disorders in children and adolescents (American Psychiatric Association, 2013). Recent epidemiological studies indicate that 25%–35% of infants in general pediatric clinics suffer from restrictive feeding difficulties, with approximately 2% progressing to anorexia (Cascales, 2017). Over the past decade, there has been a continuous decline in developmental delay among children. However, 148.1 million (22.3% of the global total) children aged < 5 years were still affected by developmental delay by 2022, with 52% of these cases in Asia and 43% in Africa (United Nations Children’s Fund et al., 2023). IA can lead to malnutrition, reduced immunity, growth retardation, and various secondary infections, thus imposing a significant burden on society (Lee et al., 2022). The pathogenesis of IA remains incompletely elucidated. It is related to environmental, pharmacological, physiological, and psychological factors. Possible causes include improper feeding methods, infections, deficiencies in micronutrient, and dysregulation of neuroendocrine (Zhao and Lin, 2024). Western medicine often utilizes symptomatic treatments such as promoting gastric motility, supplementing micronutrients, and regulating the gut microbiota. Nevertheless, relapse is common once treatment stops, and sustained benefit is elusive (He et al., 2024). Commercial Chinese polyherbal preparations (CCPPs) for treating IA center on a holistic approach, following the principle of syndrome differentiation and treatment. It employs a multi-layered and multi-target intervention model to regulate the infants’ bodily functions. This therapeutic system has a low incidence of adverse reactions and higher safety. Also, it improves symptoms in the short term and regulates physical constitution in the long term, leading to high clinical compliance. The distinct advantages of CCPPs in enhancing children’s feeding behavior and boosting nutritional metabolism are becoming more evident. It has become an important research direction for scholars at home and abroad to explore non-pharmacological alternatives for treating IA (Zeng et al., 2024).

CCPPs for treating IA have gradually attracted the attention of clinical researchers. Advantages of CCPPs lie in their convenience and safety (easy-to-take dosage forms, fewer side effects owing to their natural ingredients) as well as comprehensive therapeutic potential (uniqueness of syndrome differentiation and treatment, symptom relief and constitution regulation). Their clinical efficacy has been widely validated through practice (Liu, 2024). Several studies of clinical trials on the efficacy and safety of CCPPs in treating IA have been published. However, the differences in efficacy and safety among CCPPs remain underexplored. Conventional single randomized controlled trials (RCTs) are restricted to head-to-head comparisons. Consequently, it is difficult to comprehensively evaluate multiple CCPPs. This study employed a Bayesian network meta-analysis (BNMA) to integrate fragmented clinical evidence and enable both direct and indirect comparisons among various CCPPs. This approach overcomes the limitations of individual small-sample RCTs, clarifies the relative efficacy and safety of each CCPP, and identifies the optimal intervention strategy. Ultimately, these findings provide robust evidence-based support for standardized clinical decision-making and future related research. Nevertheless, inherent limitations in the current evidence must be noted. First, there is considerable compositional homogeneity among similar CCPPs, along with a lack of standardized dosing regimens and treatment durations. Second, individual studies often suffer from small sample sizes and insufficient evidence regarding long-term safety. Finally, variations in quality control and manufacturing processes among different pharmaceutical companies may introduce heterogeneity in clinical efficacy.

## 2 Methods

### 2.1 Study registration

Our study was implemented based upon the Preferred Reporting Items for Systematic Reviews and Meta-Analyses incorporating Network Meta-Analyses (PRISMA-NMA) guideline and was prospectively registered in PROSPERO (ID: CRD42024535550).

### 2.2 Eligibility criteria

Inclusion criteria were outlined below: (i) The study population comprised patients with a confirmed diagnosis of IA, without restrictions on race, nationality, gender, age, or disease duration; (ii) Intervention was the use of CCPP; (iii) Comparison was the control medications; (iv) Outcome measures were: (a) clinical overall effective rate, the criteria for evaluating therapeutic efficacy were established with reference to recognized standards such as Criteria of Diagnosis and Therapeutic Effect of Diseases and Syndromes in TCM (National Administration of Traditional Chinese Medicine (NATCM), 1994) and Guiding Principles for Clinical Research of New Chinese Medicines (State Food and Drug Administration (SFDA), 2002). Specifically, clinical efficacy was classified into four grades: clinically cured, markedly effective, effective, and ineffective. “Clinically cured” was defined as the pediatric patient’s appetite and food intake returning to age-appropriate normal levels, with a complete resolution of anorexia symptoms. ‘Markedly effective’ indicated a significant improvement in appetite and food intake, with the majority of the main clinical symptoms alleviated. ‘Effective’ referred to a partial improvement in appetite and symptoms compared to baseline. ‘Ineffective’ denoted no improvement or even an exacerbation of symptoms. The overall effective rate was calculated as: (number of clinically cured cases + markedly effective cases + effective cases)/total number of cases × 100%; (b) changes in weight; (c) levels of hemoglobin (Hb); (d) adverse reactions. (v) Study design was randomized controlled trials (RCTs).

Exclusion criteria were outlined below: (i) duplicates (including papers with the same data but different article types or languages; only the higher-quality ones were included); (ii) studies involving combination therapy; (iii) studies with obvious errors in trial design or data; (iv) studies using hospital-made formulations or unapproved CCPPs; (v) conference abstracts not peer-reviewed; (vi) studies derived from the same RCT; only the study with the largest sample size, most complete follow-up, and most outcome measures was included).

### 2.3 Data sources and search strategy

A search was implemented throughout CNKI, Wanfang, VIP, China Biology Medicine, PubMed, EMbase, Cochrane Library, and Web of Science for RCTs on CCPPs in treating IA up to 9 April 2024. Search terms included anorexia, infants, children, pediatrics, capsules, granules, injections, oral solutions, and pills. The search was implemented utilizing a blend of subject headings and free terms.

### 2.4 Study selection

The gathered studies were incorporated into EndNoteX9. Duplicates were identified by automated deduplication software and manual verification. After deduplication, we scrutinized titles and abstracts to exclude non-eligible studies. Complete texts were then downloaded and further reviewed to identify those that met the inclusion criteria in this analysis. The whole procedure was implemented independently by two researchers (RC Liu and JH Liu). Afterward, the results were cross-checked. Any divergences were figured out by consulting a third researcher (LR Li).

### 2.5 Data extraction

Two researchers (RC Liu and JH Liu) designed a data extraction form and independently extracted the data. The content included: (i) basic information encompassing title, author, year, study type, diagnostic criteria, interventions, treatment duration, and outcome measures; (ii) characteristics of population encompassing sample size, age and gender; (iii) methodological information encompassing randomization method, allocation concealment and blinding. Any divergences were figured out through discussion or consulting a third researcher.

### 2.6 Risk of bias in studies

Two researchers (RC Liu and JH Liu) estimated the risk of bias utilizing the Cochrane Risk of Bias tool for RCTs. This assessment tool included the following seven themes: generation of random sequence, allocation concealment, blinding of participants and intervention providers, blinding of outcome assessors, incomplete outcome data, selective outcome reporting, and other sources of bias. Each theme was assessed as a low, high, or unclear risk of bias. The results of the risk of bias assessment were visually presented utilizing Revman 5.4.

### 2.7 Synthesis methods

This study was conducted within a Bayesian analytical framework. For binary outcomes, a binomial distribution was assumed, and the model was constructed using a logit link function. For continuous outcomes, a normal likelihood function with an identity link was applied. Markov chain Monte Carlo (MCMC) methods were used for model estimation. Non-informative priors, specifically Normal (0, 10^4^), were assigned to both the study-specific baseline effects and the relative treatment effects. The between-study heterogeneity standard deviation (τ) was assigned a Uniform (0, 5) prior. Additionally, a sensitivity analysis using a Half-Cauchy prior was conducted. The results remained consistent, thereby confirming the robustness of the prior specifications. The MCMC procedure was run with 4 parallel chains, each with 50000 iterations. The first 20000 iterations were discarded as burn-in, and a thinning interval of 10 was applied. For all parameters, the Gelman–Rubin potential scale reduction factor was <1.05, and the effective sample size was >400. Trace plots and autocorrelation plots indicated good chain mixing and satisfactory control of autocorrelation, demonstrating reliable convergence of the model. This study adhered to the transitivity assumption of network meta-analysis. Through strict inclusion and exclusion criteria, baseline characteristics of the study populations, intervention durations, and criteria of outcome assessment were standardized across studies. This approach ensured a balanced distribution of effect modifiers across indirect comparisons, fulfilling the fundamental premise of transitivity. With respect to consistency, model fit was assessed using the deviance information criterion (DIC) and posterior predictive *P*-values. The difference in DIC between the consistency model and the inconsistency model was <3, suggesting no evident global inconsistency in the overall network. If closed loops were present in the network, the node-splitting method was applied to assess local consistency. Differences in treatment duration were identified as a key factor contributing to the overall study heterogeneity. To determine whether the efficacy of CCPPs and the routine drugs (RD) differed significantly according to the duration of therapy, this study specifically performed subgroup analyses based on this key variable. Treatment duration was categorized into 3 subgroups: short-term (7–21 days), medium-term (28–29 days), and long-term (30–90 days). The stratification of treatment duration into three groups was determined based on the clinical characteristics of traditional Chinese medicine for IA, the homogeneity requirements of the included studies, and standards for clinical practice. Specifically, the short-term (7–21 days) group represented short-term interventions for mild anorexia, while the long-term (30–90 days) group represented long-term management for severe cases. The medium-term (28–29 days) group was designated as a separate group because the majority of the included trials utilized a standardized 4-week intervention protocol, and this specific timeframe exhibited significant differences in efficacy and safety compared to the adjacent groups. BNMA models were fitted separately within each subgroup to compare the differences in effect sizes between CCPPs and RD across different treatment durations and to clarify the impact of treatment duration on the pooled effect. These findings indicated that the conclusions of this study should be interpreted in conjunction with the specific treatment duration to avoid overinterpretation of the efficacy data. In addition, interventions were ranked according to the surface under the cumulative ranking curve (SUCRA), and ranking plots were generated to compare differences in efficacy among the various interventions.

Multi-arm studies were implemented for the same CCPPs with different treatment durations among the included studies. Therefore, a Bayesian network meta-regression analysis was implemented to estimate whether there were significant distinctions in the effects of CCPPs with different treatment durations compared to placebo. The analysis was implemented utilizing Stata 15.0 (Stata Corporation, College Station, TX) and R4.2.0 (R development Core Team, Vienna, http://www.R-project.org). P < 0.05 denoted statistical significance.

## 3 Results

### 3.1 Study selection

**FIGURE 1:**
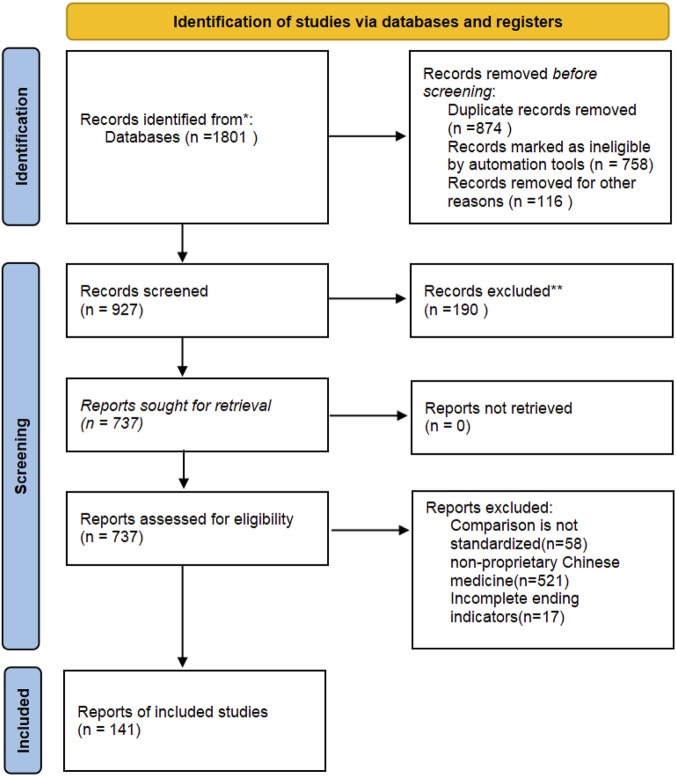
Process of literature selection.

Initially, 1801 relevant articles were identified from databases. 874 duplicates were removed. 737 articles were retained by reviewing titles and abstracts. Complete texts were analyzed to exclude 596 articles. Ultimately, 141 studies were included. The process of literature selection is provided in Figure 1.

### 3.2 Study characteristics

In total, 141 studies involving 16,963 patients and 49 types of CCPPs were included. The authors were from China. The studies were published between 1995 and 2024, with the majority appearing in the past 5 years. The specific included studies are shown in Supplementary Table S1. The diagnostic criteria for IA were primarily based upon the standards in Zhu Futang Practice of Pediatrics. The criteria included: (i) Infants refused to consume adequate food; (ii) Infants refused to eat during self-feeding or feeding; (iii) Infants exhibited absence of hunger, lacking interest in food; (iv) Infants exhibited significant growth retardation, weight loss or failure to gain weight; (v) Anorexia or food refusal caused by other diseases was excluded; (vi) Duration of the condition was greater than 1 month; (vii) Infants had a history of improper feeding or poor appetite habits.

### 3.3 Risk of bias in studies

**FIGURE 2:**
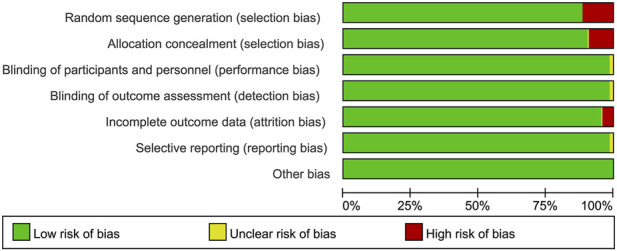
Risk of bias graph of included studies.

Regarding the generation of random sequence, six studies were assessed as unclear risk of bias. One study applied the voluntary principle, four studies allocated based upon various treatment regimens, seven studies allocated based upon the time of visit, and one study performed equal distribution among the patients. These twelve studies were rated as high risk of bias. The remaining studies leveraged random allocation and were assessed as low risk of bias. None of the studies reported on blinding, allocation concealment, selective reporting, or other sources of bias. All studies reported complete data. The risk of bias of the included studies is provided in Figure 2. The summary of the risk of bias assessment revealed that most studies lacked adequate allocation concealment, and very few implemented double-blinding or blinded outcome assessment. Consequently, performance bias and detection bias may be introduced. Such studies with a high risk of bias tended to overestimate treatment effects, potentially compromising the reliability of the overall pooled effect sizes and intervention rankings.

### 3.4 Publication bias

**FIGURE 3:**
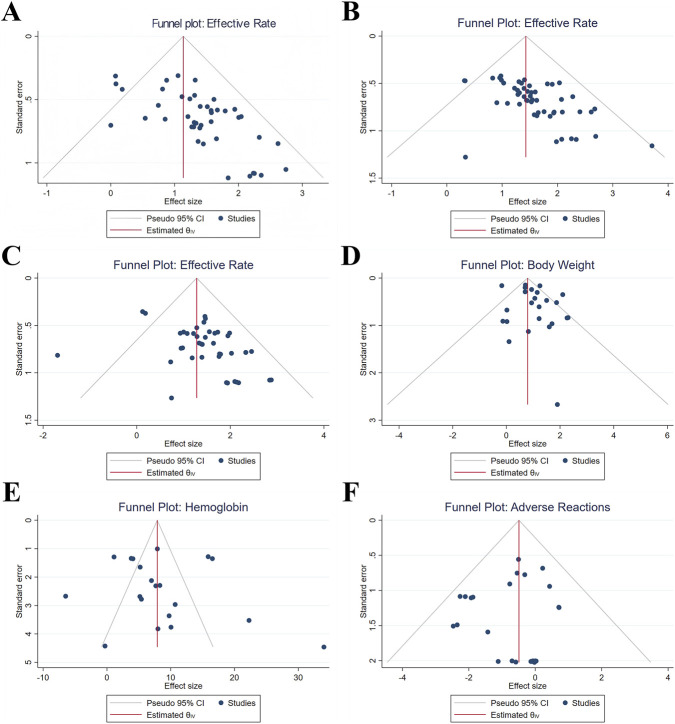
Funnel plots for assessment of publication bias of included studies. **(A)** Funnel plot for publication bias of effective rate at 7–21 days; **(B)** Funnel plot for publication bias of effective rate at 28 days; **(C)** Funnel plot for publication bias of effective rate at 30–90 days; **(D)** Funnel plot for publication bias of body weight change; **(E)** Funnel plot for publication bias of hemoglobin level; **(F)** Funnel plot for publication bias of adverse reactions.

**FIGURE 4:**
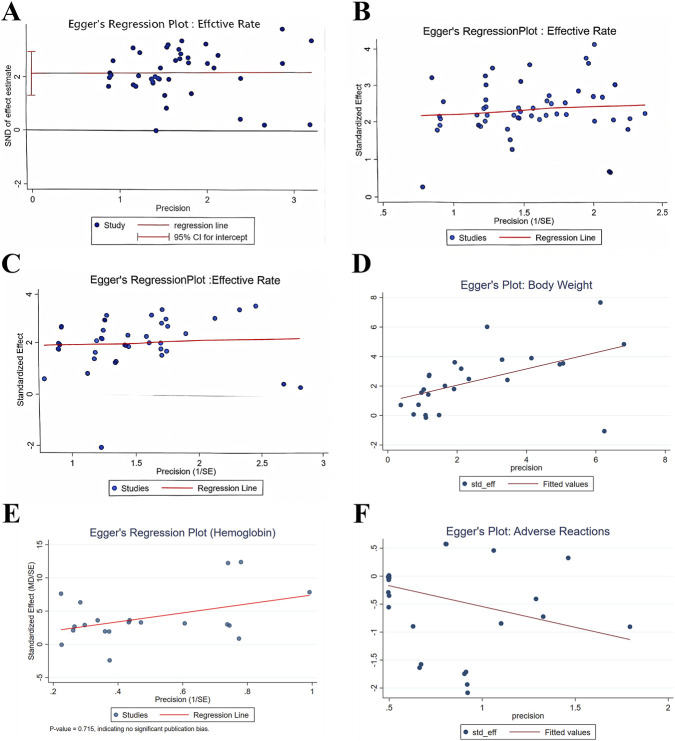
Egger’s regression plot for assessment of publication bias of included studies. **(A)** Egger’s regression plot for the effective rate at 7–21 days; **(B)** Egger’s regression plot for the effective rate at 28 days; **(C)** Egger’s regression plot for the effective rate at 30–90 days; **(D)** Egger’s regression plot for body weight change; **(E)** Egger’s regression plot for hemoglobin level; **(F)** Egger’s regression plot for adverse reactions.

Publication bias for the primary outcomes was assessed using visual inspection of funnel plots and Egger’s linear regression test. The specific results were as follows: As shown in Figures 3A–C (Funnel plots for Effective Rate Subgroups 1, 2, and 3), the distribution of studies appeared asymmetrical for the overall effective rate. This was statistically confirmed by Egger’s test, which indicated significant small-study effects (P < 0.001). This observation suggested potential publication bias, likely due to the preferential publication of trials with positive results. Regarding changes in weight, levels of Hb, and unfavorable reactions (Figures 3D–F), although visual inspection suggested some asymmetry, results of Egger’s test were non-significant (P > 0.05). This indicated no systematic difference in effect sizes between small and large studies, suggesting that the pooled effect sizes were relatively reliable. Detailed results of the Egger’s linear regression tests are presented in Figure 4.

### 3.5 Meta-analysis

#### 3.5.1 Overall clinical effective rate

##### 3.5.1.1 Associations between interventions

**FIGURE 5:**
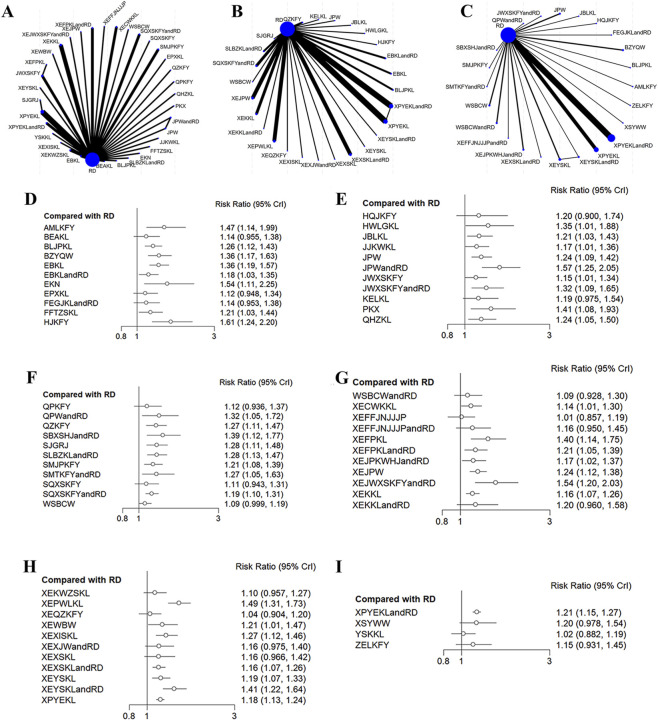
**(A)** Network of the effective rate of commercial Chinese polyherbal preparations in treating infantile anorexia (7–21 days); **(B)** Network of the effective rate of commercial Chinese polyherbal preparations in treating infantile anorexia (28 days); **(C)** Network of the effective rate of commercial Chinese polyherbal preparations in treating infantile anorexia (30–90 days); **(D,E)** Forest plot of the meta-analysis on the effective rate of commercial Chinese polyherbal preparations relative to the control medications ((7–21 days)); **(F,G)** Forest plot of the meta-analysis on the effective rate of commercial Chinese polyherbal preparations relative to the control medications (28 days); **(H,I)** Forest plot of the meta-analysis on the effective rate of commercial Chinese polyherbal preparations relative to the control medications (30–90 days).

This study implemented a BNMA based upon 141 clinical studies on CCPPs in treating IA, involving 49 types of CCPPs. For clarity, these CCPPs were labeled with their Pinyin name plus dosage forms in the text, and with Pinyin initials in the figures, as detailed in Table 1. To improve the readability of the graphs, the overall network was classified into three subgroups based on intervention duration. Figure 5A presents the network for short-term treatments (7–21 days). Figure 5B focuses on the comparison of standard 28-day treatments. Figure 5C systematically demonstrates the relationships between medium- and long-term treatments (30–90 days). In clinical practice, the findings of this study should be interpreted in conjunction with treatment duration to avoid overinterpretation of the efficacy data. This layered presentation ensured both the recognition of graphs and clearly reflected comparative associations between interventions across various treatment durations. This research identified three closed loops: (Jianpi Pills [JPW], JPW and RD); (Xingpi Yang’er Granule, Xingpi Yang’er Granule and RD); and (XE Yanshi Granule, XE Yanshi Granule and RD). Direct comparisons between Xingpi Yang’er Granules and the control medications were the most common.

**TABLE 1:** Abbreviations in images, simplified names in the text, and full English names.

No.	Abbreviation	Simplified name	Full English name
1	AMLKFY	Anmole OS	Anmole oral solution
2	BEAKL	Baoer’an granule	Baoer’an granules
3	BLJPKL	Bailing jianpi granule	Bailing jianpi granules
4	BZYQW	Buzhong yiqi pill	Buzhong yiqi pills
5	EBKL	Erbao granule	Erbao granules
6	EKN	Erkangning	Erkangning
7	EPXKL	Erpixing granule	Erpixing granules
8	FEGJKL	Feier ganji granule	Feier ganji granules
9	FEGJKLandRD	Feier ganji granules + RD	Feier ganji granules plus routine drugs
10	FFTZSKL	Fufang taizishen granule	Fufang taizishen granules
11	HJKFY	Huaji OS	Huaji oral solution
12	HQJKFY	Huangqijing OS	Huangqijing oral solution
13	HWLGKL	Hewei liaogan granule	Hewei liaogan granules
14	JBLKL	Jianbaoling granule	Jianbaoling granules
15	JJKWKL	Jinju kaiwei granule	Jinju kaiwei granules
16	JPW	Jianpi pill	Jianpi pills
17	JPWandRD	Jianpi pill + RD	Jianpi pills plus routine drugs
18	JWXSKFY	Jianwei xiaoshi OS	Jianwei xiaoshi oral solution
19	JWXSKFYandRD	Jianwei xiaoshi OS + RD	Jianwei xiaoshi OS plus routine drugs
20	KELKL	Kangerling granule	Kangerling granules
21	PKX	Pikexin	Pikexin
22	QHZKL	Qihuozha granule	Qihuozha granules
23	QPKFY	Qipi OS	Qipi oral solution
24	QPW	Qipi pill	Qipi pills
25	QPWandRD	Qipi pill + RD	Qipi pills plus routine drugs
26	QZKFY	Qizao OS	Qizao oral solution
27	SBXSHJ	Shanbai xiaoshi mixture	Shanbai xiaoshi mixture
28	SBXSHJandRD	Shanbai xiaoshi mixture + RD	Shanbai xiaoshi mixture plus routine drugs
29	SJGRJ	Shaji dry emulsion	Shaji dry emulsion
30	SLBZKL	Shenling baizhu granule	Shenling baizhu granules
31	SLBZKLandRD	Shenling baizhu granule + RD	Shenling baizhu granule plus routine drugs
32	SMJPKFY	Shanmai jianpi OS	Shanmai jianpi oral solution
33	SMTKFY	Simo tang OS	Simo tang oral solution
34	SMTKFYandRD	Simo tang OS + RD	Simo tang oral solution plus routine drugs
35	SQXSKFY	Shenqu xiaoshi OS	Shenqu xiaoshi oral solution
36	SQXSKFYandRD	Shenqu xiaoshi OS + RD	Shenqu xiaoshi OS plus routine drugs
37	WSBCW	Wangshi baochi pill	Wangshi baochi pills
38	WSBCWandRD	Wangshi baochi pill + RD	Wangshi baochi pill plus routine drugs
39	XECWKKL	XECW granule	Xiaoer changweikang granules
40	XEFFJNJJJP	XEFFJ chewable tablet	Xiaoer fufang jineijin chewable tablets
41	XEFPKL	XE fupi granule	Xiaoer fupi granules
42	XEJPKWHJ	XEJP kaiwei mixture	Xiaoer jianpi kaiwei mixture
43	XEJPW	XE jianpi pill	Xiaoer jianpi pills
44	XEJWXSKFY	XE jianwei xiaoshi OS	Xiaoer jianwei xiaoshi oral solution
45	XEKKL	XE kang granule	Xiaoer kang granules
46	XEKWZSKL	XEKW zengshi granule	Xiaoer kaiwei zengshi granules
47	XEPWLKL	XE piweile granule	Xiaoer piweile granules
48	XEQZKFY	XE qizha OS	Xiaoer qizha oral solution
49	XEWBW	XE weibao pill	Xiaoer weibao pills
50	XEXISKL	XE xishi granule	Xiaoer xishi granules
51	XEXJW	XE xiangju pill	Xiaoer xiangju pills
52	XEXSKL	XE xiaoshi granule	Xiaoer xiaoshi granules
53	XEYSKFY	XE yanshi OS	Xiaoer yanshi oral solution
54	XEYSKL	XE yanshi granule	Xiaoer yanshi granules
55	XPYEKL	Xingpi Yang’er granule	Xingpi Yang’er granules
56	XSYWW	Xiangsha yangwei pill	Xiangsha yangwei pills
57	YSKKL	Yanshi kang granule	Yanshi kang granules
58	ZELKFY	Zhangerling OS	Zhangerling oral solution

##### 3.5.1.2 Synthesized results

The results of the BNMA displayed that Huaji Oral Solution (Huaji OS) (RR: 1.61, 95% CI: 1.24–2.20), Jianpi Pill + RD (RR: 1.57, 95% CI: 1.25–2.05) and Erkangning (RR: 1.54, 95% CI: 1.11–2.25) had a significantly higher overall effective rate relative to the control medications (P < 0.05). Detailed data are shown in Figures 5D–I. Significant differences were observed in the overall effective rate between some CCPPs [Huaji OS: RR = 1.62, 95%CI (1.24, 2.19), P < 0.05; Jianpi Pill + RD: RR = 1.57, 95%CI (1.25, 2.06), P < 0.05; Erkangning: RR = 1.54, 95%CI (1.10, 2.27), P < 0.05]. Detailed data are shown in Supplementary Table S2; Supplementary Table S3. Given the volume of data generated in this study, only the most representative figures are presented in the main text to maintain conciseness. Extended results are provided in Supplementary Tables S4–S7. Based on the rankings of SUCRA, the top three measures were Huaji OS (0.92), Jianpi Pill + RD (0.91), XE Piweile Granules (0.89). According to the SUCRA ranking, RD exhibited the lowest cumulative ranking probability (0.04) among the included trials, indicating that CCPPs demonstrated statistically superior efficacy in improving the overall response rate compared to conventional treatment. Detailed data are shown in Table 2.

**TABLE 2:** Bayesian network meta-analysis of rankings and rank probabilities for each commercial Chinese polyherbal preparation based on the overall effective rate.

Interventions	Effective rate	
	SUCRA	Rank
HJKFY	0.92	1
JPWandRD	0.91	2
XEPWLKL	0.89	3
XEJWXSKFYandRD	0.88	4
EKN	0.85	5
AMLKFY	0.83	6
XEYSKLandRD	0.82	7
XEFPKL	0.79	8
SBXSHJandRD	0.77	9
EBKL	0.77	10
PKX	0.77	11
BZYQW	0.76	12
JWXSKFYandRD	0.69	13
HWLGKL	0.69	14
QPWandRD	0.67	15
SLBZKLandRD	0.64	16
SJGRJ	0.63	17
XEXISKL	0.62	18
QZKFY	0.61	19
SMTKFYandRD	0.61	20
BLJPKL	0.59	21
XEJPW	0.56	22
JPW	0.55	23
QHZKL	0.55	24
SMJPKFY	0.50	25
XEWBW	0.49	26
XPYEKLandRD	0.49	27
FFTZSKL	0.49	28
JBLKL	0.48	29
XEFPKLandRD	0.48	30
XSYWW	0.47	31
HQJKFY	0.47	32
XEKKLandRD	0.47	33
KELKL	0.46	34
SQXSKFYandRD	0.44	35
XEYSKL	0.43	36
XPYEKL	0.40	37
EBKLandRD	0.40	38
XEJPKWHJandRD	0.40	39
JJKWKL	0.38	40
XEFFJNJJJPandRD	0.38	41
XEXJWandRD	0.38	42
XEXSKL	0.37	43
ZELKFY	0.37	44
JWXSKFY	0.35	45
XEKKL	0.35	46
XEXSKLandRD	0.34	47
FEGJKLandRD	0.33	48
BEAKL	0.33	49
XECWKKL	0.31	50
EPXKL	0.29	51
QPKFY	0.29	52
SQXSKFY	0.26	53
XEKWZSKL	0.23	54
WSBCWandRD	0.23	55
WSBCW	0.18	56
XEQZKFY	0.12	57
YSKKL	0.10	58
XEFFJNJJJP	0.09	59
RD	0.04	60

##### 3.5.1.3 Meta-regression

To explore the effect of treatment duration on overall effective rate, a meta-regression analysis was performed on the duration differences among the included studies. The analysis displayed that the overall effective rate of CCPPs in treating IA was not significantly correlated to treatment duration relative to the control medications, with no statistical significance. Detailed data are shown in Supplementary Table S8.

#### 3.5.2 Changes in weight

##### 3.5.2.1 Associations between interventions

**FIGURE 6:**
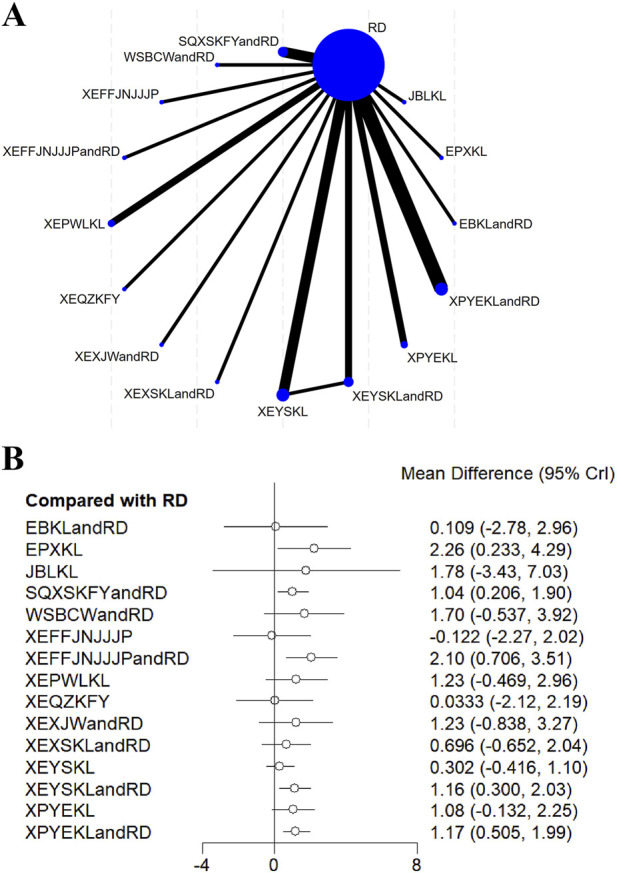
**(A)** Network of commercial Chinese polyherbal preparation treatments for changes in weight in infantile anorexia; **(B)** Forest plot of the meta-analysis on the efficacy of commercial Chinese polyherbal preparations for changes in weight relative to the control medications.

Twenty-four studies reported changes in weight, involving 12 types of CCPPs. Specifically, these included XE Xiaoshi Granule, Xingpi Yang’er Granule, XE Yanshi Granule, XE Piweile Granule, Erbao Granule, XE Qizha OS, XE Xiangju Pills, Jianbaoling Granule, XEFFJ Chewable Tablet, Erpixing Granule, Shenqu Xiaoshi OS, and Wangshi Baochi Pill. One closed loop was detected, which was (XE Yanshi Granule, XE Yanshi Granule + RD). The direct comparisons between Xingpi Yang’er Granule and the control medications were the most frequent. Detailed data are shown in Figure 6A.

##### 3.5.2.2 Synthesized results

The results of the BNMA showed that Erpixing Granule [MD = 2.26, 95%CI (0.23, 4.29)], XEFFJ Chewable Tablet combined with RD (MD = 2.1, 95%CI (0.71, 3.51); P < 0.05) had significant advantages in improving patients’ weight compared with the control medications, while Jianbaoling Granule [MD = 1.78, 95%CI (−3.43, 7.03)] showed no statistical significance in improving patients’ weight relative to the control medications since its 95% confidence interval crossed 0. Detailed data are shown in Figure 6B. There were differences in the effectiveness of some CCPPs in improving weight [Erpixing Granule: MD = 2.26, 95% CI (0.23, 4.29); XEFFJ Chewable Tablet + RD: MD = 2.1, 95% CI (0.71, 3.51), P < 0.05]. Detailed data are shown in Supplementary Table S9. Extended results are provided in Supplementary Table S10. Based on the rankings of SUCRA, the top three measures were XEFFJ Chewable Tablet + RD (0.83), Erpixing Granule (0.82) and Wangshi Baochi Pill + RD (0.69). Detailed data are shown in Table 3. Based on the SUCRA rankings, RD had the lowest probability of being the optimal treatment (SUCRA = 0.16). This suggested that CCPPs demonstrated a greater improvement in body weight compared to conventional therapy.

**TABLE 3:** Bayesian network meta-analysis of rankings and rank probabilities for each commercial Chinese polyherbal preparation based upon changes in weight.

Interventions	Changes in weight	
	SUCRA	Rank
EPXKL	0.83	1
XEFFJNJJJPandRD	0.82	2
WSBCWandRD	0.69	3
JBLKL	0.62	4
XPYEKLandRD	0.59	5
XEPWLKL	0.58	6
XEYSKLandRD	0.58	7
XEXJWandRD	0.57	8
XPYEKL	0.55	9
SQXSKFYandRD	0.53	10
EBKLandRD	0.31	11
XEQZKFY	0.26	12
XEFFJNJJJP	0.22	13
RD	0.16	14

##### 3.5.2.3 Meta-regression

To explore the impact of treatment duration on changes in weight, a meta-regression analysis on the differences of duration was implemented among the included studies. The analysis displayed that the effects of CCPPs for changes in weight were not significantly correlated to treatment duration relative to the control medications, with no statistical significance. Detailed data are shown in Supplementary Table S11.

#### 3.5.3 Levels of hb

##### 3.5.3.1 Associations between interventions

**FIGURE 7:**
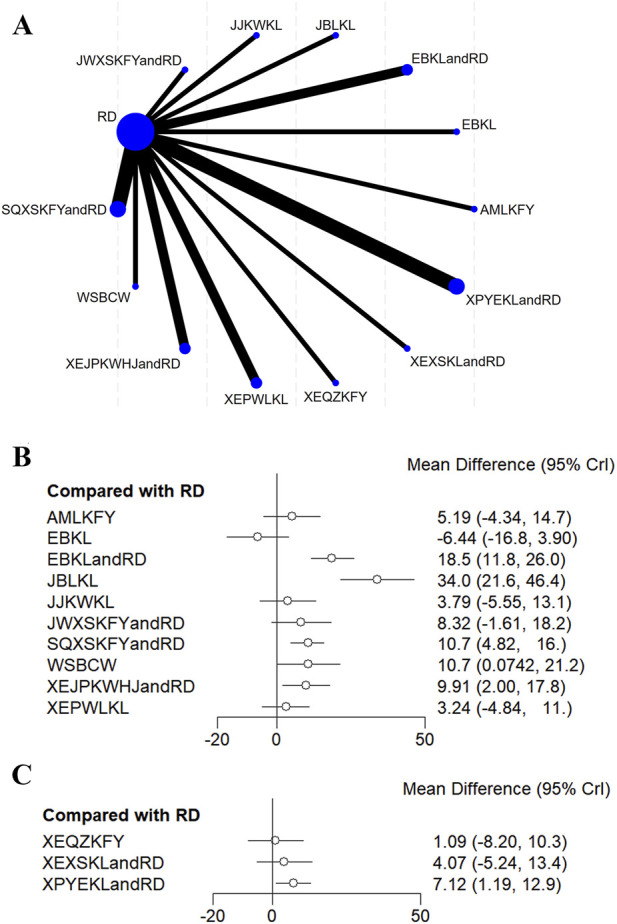
**(A)** Network of commercial Chinese polyherbal preparation treatments for infantile anorexia based upon the level of hemoglobin; **(B,C)** Forest plot of the meta-analysis on the efficacy of commercial Chinese polyherbal preparations for hemoglobin relative to the control medications.

Twenty studies reported levels of Hb, involving 12 types of CCPPs. Specifically, these included Jianbaoling Granule, Erbao Granule, Shenqu Xiaoshi OS, Wangshi Baochi Pill, XEJP Kaiwei Mixture, Jianwei Xiaoshi OS, Xingpi Yang’er Granule, Anmole OS, XE Xiaoshi Granules, Jinju Kaiwei Granule, XE Piweile Granule, and XE Qizha OS. Among them, studies directly comparing Xingpi Yang’er Granule + RD were the most numerous. The graph displayed no closed loops. Detailed data are shown in Figure 7A.

##### 3.5.3.2 Synthesized results

The results of BNMA displayed that CCPPs had a significant advantage in improving levels of Hb relative to the control medications, such as Jianbaoling Granule (MD = 34.0,95%CI (21.6, 46.4), Erbao Granule + RD (MD = 18.5,95%CI (11.8, 26.0) and Shenqu Xiaoshi OS + RD (MD = 10.7,95%CI (4.82, 16.0). Detailed data are shown in Figures 7B,C. There were differences in the effectiveness of some CCPPs in improving the levels of Hb [Jianbaoling Granule: MD = 33.97, 95% CI (21.55, 46.4), P < 0.05; Erbao Granule + RD: MD = 18.5, 95% CI (11.78, 26.03), P < 0.05; Shenqu Xiaoshi OS + RD: MD = 10.72, 95% CI (4.8, 16.02), P < 0.05]. Detailed data are shown in Supplementary Table S12; Supplementary Table S13. Based on the rankings of SUCRA, the top three measures were Jianbaoling Granule (1), Erbao Granule + RD (0.9) and Shenqu Xiaoshi OS + RD (0.71). According to the SUCRA ranking, RD had the second-lowest probability of being optimal (SUCRA = 0.16), ranking only above Erbao Granule. This suggested that most CCPPs were superior to conventional therapy in improving the levels of Hb. Detailed data are shown in Table 4.

**TABLE 4:** Bayesian network meta-analysis of rankings and rank probabilities for each commercial Chinese polyherbal preparation based upon levels of hemoglobin.

Interventions	Levels of hb	
	SUCRA	Rank
JBLKL	1	1
EBKLandRD	0.9	2
SQXSKFYandRD	0.71	3
WSBCW	0.68	4
XEJPKWHJandRD	0.66	5
JWXSKFYandRD	0.58	6
XPYEKLandRD	0.53	7
AMLKFY	0.43	8
XEXSKLandRD	0.38	9
JJKWKL	0.36	10
XEPWLKL	0.33	11
XEQZKFY	0.24	12
RD	0.16	13
EBKL	0.04	14

##### 3.5.3.3 Meta-regression

A meta-regression analysis was implemented to examine the effect of treatment duration on levels of Hb. The results showed that the effect of CCPPs on levels of Hb was not significantly associated with treatment duration relative to the control medications, with no statistical significance. Detailed data are shown in Supplementary Table S14.

#### 3.5.4 Adverse reactions

##### 3.5.4.1 Associations between interventions

**FIGURE 8:**
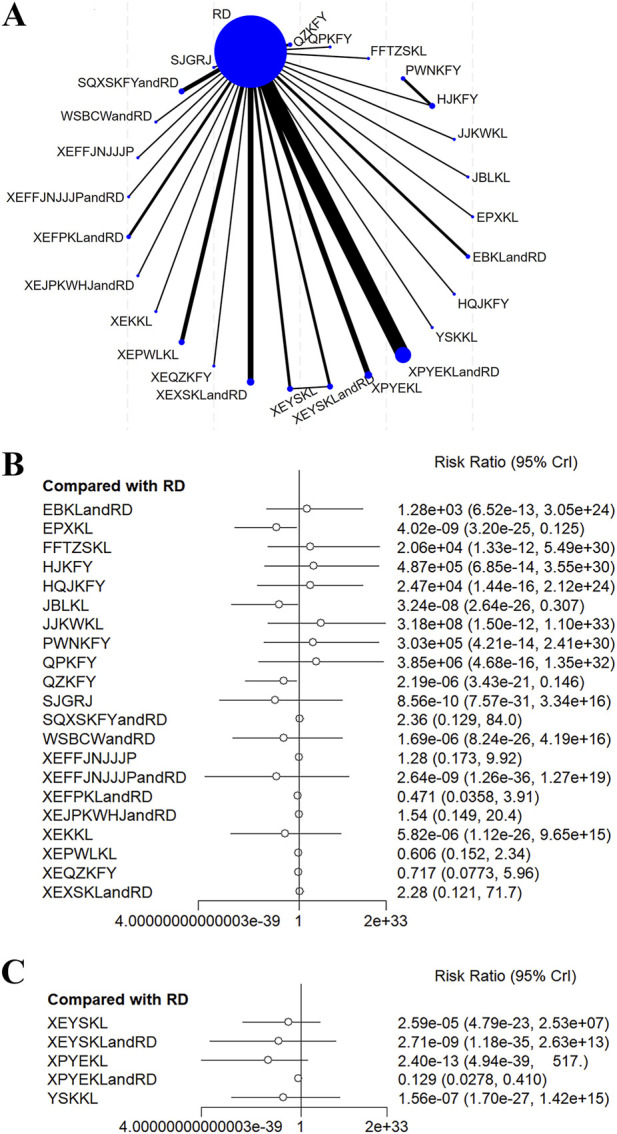
**(A)** Network of commercial Chinese polyherbal preparation treatments for infantile anorexia based upon adverse reactions; **(B,C)** Forest plot of the meta-analysis on adverse reactions in commercial Chinese polyherbal preparation treatments for infantile anorexia relative to the control medications.

Forty-nine studies reported adverse reactions, involving 22 types of CCPPs. Specifically, these included XE Xiaoshi Granule, XEJP Kaiwei Mixture, Xingpi Yang’er Granule, Shenqu Xiaoshi OS, XE Kang Granule, XE Yanshi Granule, XE Piweile Granule, Erbao Granule, Wangshi Baochi Pill, XE Qizha OS, XE Fupi Granule, Huaji OS, Huangqijing OS, Jianbaoling Granule, Jinju Kaiwei Granule, Qipi OS, Qizao OS, Shaji Dry Emulsion, XEFFJ Chewable Tablet, Yanshi Kang Granule, Fufang Taizishen Granule, and Erpixing Granule. One closed loop was observed, which was (XE Yanshi Granule, XE Yanshi Granule + RD). The direct comparisons between Xingpi Yang’er Granule and the control medications were the most common. Detailed data are shown in Figure 8A.

##### 3.5.4.2 Synthesized results

The safety of different CCPPs in treating IA was not statistically significant relative to the control medications. However, Jinju Kaiwei Granule had more adverse events (RR = 3.18e+08 95% CI (1.5e-12, 1.1e+33), P < 0.05) relative to the control medications. Detailed data are shown in Figures 8B,C. Based on the rankings of SUCRA, the top three measures were Xingpi Yang’er Granule (0.82), Erpixing Granule (0.8) and Jianbaoling Granule (0.78). The RD ranked 16th (SUCRA = 0.40), suggesting that numerous CCPP interventions were associated with a lower risk of adverse reactions. Detailed data are shown in Table 5. The results showed that the safety of CCPPs had no significant correlation with treatment duration relative to the control medications, with no statistical significance. Detailed data are shown in Supplementary Table S15. Specific adverse reactions are provided in Table 6.

**TABLE 5:** Bayesian network meta-analysis of rankings and rank probabilities for commercial Chinese polyherbal preparations based upon adverse reactions.

Interventions	Adverse reactions	
	SUCRA	Rank
XPYEKL	0.82	1
EPXKL	0.80	2
JBLKL	0.78	3
QZKFY	0.75	4
XEYSKLandRD	0.68	5
YSKKL	0.68	6
SJGRJ	0.68	7
XEFFJNJJJPandRD	0.64	8
WSBCWandRD	0.61	9
XEYSKL	0.61	10
XEKKL	0.60	11
XPYEKLandRD	0.58	12
XEFPKLandRD	0.48	13
XEPWLKL	0.46	14
XEQZKFY	0.44	15
RD	0.40	16
XEFFJNJJJP	0.38	17
XEJPKWHJandRD	0.37	18
XEXSKLandRD	0.34	19
EBKLandRD	0.34	20
SQXSKFYandRD	0.34	21
FFTZSKL	0.32	22
HQJKFY	0.31	23
PWNKFY	0.30	24
QPKFY	0.30	25
HJKFY	0.28	26
JJKWKL	0.21	27

**TABLE 6:** Adverse reactions of commercial Chinese polyherbal preparations in the treatment of infantile anorexia.

Interventions	Number of research	Group	N/case	Adverse reactions
EBKL + RDvsRD	2	T	116	No
	​	C	116	No
EPXKLvsRD	1	T	100	3 cases of nausea and rash
	​	C	100	5 cases involving thrombocytopenia, dizziness, vomiting, and urinary tract infection
FFTZSKLvsRD	1	T	160	No
	​	C	150	No
HJKFYvsRD	1	T	56	No
	​	C	56	No
HQJKFYvsRD	1	T	35	No
	​	C	20	No
JBLKL	1	T	62	No
	​	C	61	2 cases of nausea and vomiting
JJKWKL	1	T	121	No
	​	C	122	3 cases of diarrhea
QZKFYvsRD	2	T	66	No
	​	C	66	No
QPKFYvsRD	1	T	45	No
	​	C	40	No
SJGRJvsRD	1	T	80	No
	​	C	80	No
SQXSKFY + RDvsRD	3	T	134	1 mild rash and 1 case of diarrhea
	​	C	134	1 case of edema
WSBCWvsRD	1	T	21	No
	​	C	21	No
XEFPKL + RDvsRD	2	T	122	1 case of vomiting and 1 case of nausea
	​	C	125	2 cases of vomiting, 1 case of constipation, and 1 case of nausea
XEFFJNJJJP + RDvsRD	2	T	155	1 case of nausea and 1 case of vomiting
	​	C	154	5 cases of dizziness, vomiting, and urticaria
XEJPKWHJ + RDvsRD	1	T	40	3 cases of diarrhea, nausea, and rash
	​	C	40	2 cases of dizziness and nausea
XEKKL	1	T	90	No
	​	C	30	No
XEPWLKLvsRD	3	T	169	8 cases of nausea and abdominal pain
	​	C	166	14 cases of vomiting and abdominal pain
XEQZKFYvsRD	1	T	113	1 case of upper respiratory tract infection, 1 case of suppurative tonsillitis, 1 case of pancreatitis
	​	C	111	2 cases of rash, 2 cases of upper respiratory tract infection
XEXSKL + RDvsRD	4	T	240	No
	​	C	318	No
XPYEKL + RDvsRD	8	T	429	4 cases of diarrhea
	​	C	426	27 cases involving diarrhea, abdominal pain, vomiting, and rash
XPYEKLvsRD	7	T	340	No
	​	C	324	No
YSKKLvsRD	1	T	200	No
	​	C	100	No
XEYSKFYvsRD	1	T	42	No
	​	C	39	No

## 4 Discussion

The core pathogenesis of IA arises from impaired digestion and absorption by the spleen and stomach, and the liver and kidneys are concurrently affected. CCPPs focus on syndrome differentiation and treatment as well as a holistic perspective (Yang et al., 2025). TCMs markedly improve the poor appetite, small food intake, abdominal distension, and emaciation in children with IA, winning broad acceptance from both patients and their parents. Studies indicate that TCMs work through multiple mechanisms, including regulating the intestinal microbiota, influencing appetite-regulating factors, promoting secretion of digestive enzymes, adjusting secretion of gastrointestinal hormones, improving gastrointestinal motility, and enhancing the immune system of the body (He et al., 2025). Therefore, this study utilizes BNMA to compare the efficacy of oral CCPPs and conventional Western medicines in treating IA.

This study included 141 studies, covering 49 types of CCPPs and four outcome measures. Direct and indirect comparisons of the included CCPPs indicated that CCPPs were effective in treating IA. The BNMA suggested that, in terms of improving clinical effective rate, Huaji OS was the best intervention. It is composed of 10 herbal ingredients: poria (peeled), cuttlebone, roasted chicken gizzard membrane, vinegar-processed sparganium rhizome, vinegar-processed curcuma zedoary rhizome, safflower, betel nut, omphalia, carpesium fruit, and rangooncreeper fruit. It can strengthen the spleen, promote the movement of qi, and eliminate accumulation and malnutrition (Huang et al., 2013). Clinical evidence has confirmed that Huaji OS can promote gastric secretion, increase activity of gastric pepsin, improve intestinal microcirculation, and enhance the function of duodenal contraction (Wu and Teng, 2017). Studies have shown that Huaji OS can enhance the propulsion of the small intestine and promote gastric emptying of the mouse (Liu et al., 2017). Regarding changes in weight, research confirmed that Erpixing Granule mainly acted through components such as quercetin, isorhamnetin, β-sitosterol, and kaempferol, acting on the functional and inflammatory targets such as JUN, RELA, MAPK, and AKT1. It also participated in signaling pathways such as HIF-1, IL-17, TNF, and PI3K/Akt to exert anti-inflammatory effects, regulate the microbiota, combat depression and improve the clinical symptoms of affected infants (Dong et al., 2023). Studies showed that Erpixing Granule was mainly leveraged to treat IA, given that it can promote digestive function and propulsion of the small intestine, effectively increase levels of subpopulations of T cell and serum immunoglobulin, relieve pain, and treat anemia (Li et al., 2022). Regarding the levels of Hb, Jianbaoling Granule has a significant advantage. Jianbaoling Granule is composed of white fungus, poria, yam, hawthorn, and lysine. It can improve symptoms of IA through the synergistic effects of multiple components. Its mechanism covers the regulation of digestive function, control of central appetite, and immune-metabolic coordination: (i) Starch and polyphenol oxidase in yam enhance gastrointestinal digestion and absorption. The organic acids (such as hawthorn acid and malic acid) in hawthorn improve digestive efficiency by activating pepsin. Proteins and lecithin components in poria synergistically regulate the transport function of spleen and stomach; (ii) Mucoprotein in yam regulates the levels of blood sugar to act on the hunger center, while poria and lysine activate the central nervous system through metabolism of neurotransmitter and synthesis of acetylcholine. They collectively stimulate appetite-related signaling pathways; (iii) The acidic polysaccharides in white fungus enhance immune status by boosting the phagocytic activity of leukocytes, modulating lymphocyte-subset ratios, and promoting bone-marrow hematopoiesis. As an essential amino acid, lysine participates in the metabolism of pyruvate and biosynthesis of protein, promoting homeostasis of energy metabolism. Jianbaoling Granule achieves synergistic enhancement through multi-target intervention (digestion-immunity-neuroaxis), aligning with the theory of tonifying the spleen, nourishing the stomach, and strengthening the spleen and lung in CCPPs (Zhang, 2019; Chen, 2015; Huang et al., 2012). In terms of safety, Jinju Kaiwei Granule ranked significantly lower than the control drug. Also, it ranked lower than other CCPPs, including Shaji Dry Emulsion, Erpixing Granule, and Jianbaoling Granule. This may be attributed to an extreme distribution of adverse events in the included studies. It should be noted that this observation was not caused by data errors or improper selection of statistical models, but was closely related to the inherent characteristics of clinical trials of CCPPs. The adverse reactions of CCPPs are mostly mild. Symptoms include nausea, vomiting, abdominal pain, diarrhea, rashes, and infections of the upper respiratory tract. Studies have reported that patients can recover on their own or recover after treatment. In terms of drug safety (Chen et al., 2024), none of the studies reported severe adverse reactions. Adverse effects were generally well tolerated. The adverse reactions can be relieved or disappear after discontinuation of drug, adjustment in dose, or symptomatic management, indicating a favorable safety profile for CCPPs in treating IA. Results of meta-regression indicate that the treatment duration has little impact on the efficacy of CCPPs for IA.

In a clinical trial investigating the therapeutic efficacy of Huaji OS for treating IA, 112 children were randomly classified into two cohorts. The treatment cohort (56 cases) received oral Huaji OS, while the control cohort (56 cases) received a placebo medication. The results displayed that the overall effective rate of the Huaji OS cohort (89.29%) was considerably higher relative to the control cohort (55.36%). The distinction denoted statistical significance (P < 0.01). In summary, Huaji OS demonstrated significant efficacy in treating IA, effectively improving clinical symptoms, promoting appetite, and enhancing digestive and absorption functions (Wu and Wang, 2007). In a RCT involving 200 kids diagnosed with spleen deficiency syndrome and IA, Erpixing Granule showed superior efficacy in significantly improving core symptoms such as poor appetite, reduced food intake, and irregular bowel movements relative to ginseng–poria–atractylodes compound tablets (P < 0.05). Moreover, it effectively reduced meal duration and boosted body weight (P < 0.05). Meanwhile, it maintained an excellent safety profile with no serious adverse reactions reported (Liu and Wang, 2022). Previous meta-analyses and NMA indicated that Erpixing Granule effectively alleviated IA, particularly improving symptoms such as poor appetite, pale complexion, belching, and hiccups. It demonstrated superior efficacy and a more favorable safety profile relative to other drugs (Ju et al., 2023). In a RCT involving 123 children with IA due to spleen-stomach disharmony, Jianbaoling Granule significantly improved the overall effective rate (88.7% vs. 73.8%, P < 0.05) compared to the domperidone tablet cohort. It also notably improved food intake, levels of blood zinc (P < 0.05), and levels of Hb (both within-group and between-group comparisons, P < 0.05). No significant adverse reactions were observed (Li and Zhang, 2016). A RCT involving 86 children with IA exhibited that Jianbaoling Granule significantly improved the overall effective rate (88.4% vs. 72.1%, P < 0.05) compared to enzyme tablet cohort. It also exhibited a good safety profile and improved levels of trace elements such as zinc and iron, as well as immunological indices IgA, IgG, and IgM (between-group comparisons, P < 0.05) (Li and Li, 2016).

Furthermore, this study observed a certain degree of heterogeneity in efficacy, which may be related to differences in the composition and specific therapeutic targets of different CCPPs. For instance, Huaji OS and similar drugs focus on resolving food stagnation and regulating qi to stimulate appetite. They contain ingredients like roasted chicken gizzard membrane, sparganium rhizome, and codonopsis root, which can rapidly improve subjective symptoms of anorexia, thereby leading to a high overall effective rate. However, they lack specific components for promoting nutrient absorption or tonifying qi and nourishing blood, resulting in limited effects on weight gain and the levels of Hb. In contrast, drugs showing advantages in weight gain (e.g., Erpixing Granule) contain yam and lysine, focusing on invigorating the spleen, consolidating the intestines, and promoting protein synthesis. Drugs superior in improving Hb (e.g., Erbao Granule) contain pseudostellaria heterophylla and paeonia lactiflora, focusing on tonifying qi and nourishing blood. Notably, Jianbaoling Granule combines spleen-invigorating ingredients like yam with nutrients like lysine. It not only invigorates the spleen and consolidates the intestines to promote protein synthesis, thereby increasing weight. Also, it assists in the generation of qi and blood by supplementing nutrition, thus raising the levels of Hb. Consequently, it demonstrates efficacy for both metrics. These drugs target different pathological processes, resulting in differences in objective indicators and consequent heterogeneity in efficacy. Therefore, clinical selection should be tailored to the specific needs of the child. Drugs that resolve food stagnation should be prioritized for improving subjective anorexia symptoms, while those that invigorate the spleen to promote protein synthesis or tonify qi and nourish blood should be selected for increasing weight or correcting anemia, respectively. This individualized approach may help optimize clinical outcomes.

## 5 Conclusion

By systematically comparing the efficacy of various CCPPs in treating IA using a SNMA, this study offers clear value for clinical practice. First, it clarifies the optimal hierarchical order of medications: HJKFY ranked highest for overall clinical effective rate, EPXKL was the most effective for weight gain, and JBLKL was the preferred choice for elevating Hb levels. These findings enable pediatricians to rapidly select optimal formulations, thereby reducing the arbitrariness of empirical prescribing. Second, these findings are applicable to diverse disease conditions and treatment durations. Based on the subgroup analysis, short-term treatment is suitable for children with mild appetite loss, whereas long-term treatment is more appropriate for patients with persistent spleen-stomach deficiency. Consequently, our findings may provide novel insights for individualized treatment regimens. Third, CCPPs used for this study aligns with pediatric physiological characteristics. Characterized by mild pharmacological properties and high compliance of patients, CCPPs are well-suited for the delicate constitution of children. Therefore, they can serve as first-line interventions and are ideal for long-term care in primary healthcare settings and at home. Fourth, this study helps prevent polypharmacy and resource waste. By clarifying the specific efficacy of various formulations, this study discourages the overlapping use of spleen-invigorating CCPPs, thereby conserving medical resources and reducing the medication burden on children. Overall, these findings may provide evidence-based guidance for the standardized selection of CCPPs and the formulation of individualized interventions in pediatric practice, while also offering valuable insights for future clinical research regarding CCPPs in treating IA. However, these results are based upon current trial data and may not reflect actual clinical efficacy. Regarding Hb, it should be noted that the included subjects suffered from chronic anorexia and malnutrition, presenting with low baseline Hb levels and overt anemia. Consequently, the substantial improvement in Hb levels was observed following the intervention of CCPPs. Furthermore, given that Hb is a sensitive biochemical marker, its measurement can be influenced by various confounding factors. In the interpretation of clinical efficacy, it is crucial to consider the baseline conditions of pediatric patients to avoid misinterpreting the treatment outcomes based solely on the values of the effect size. Therefore, it is necessary to apply more large-scale, multi-center, high-quality RCTs to verify these findings.

## 6 Strengths and limitations

Our study has several major strengths. CCPPs for treating IA are a current research hotspot, but evidence on the differences in efficacy and safety between various CCPPs remains underexplored. A NMA of different TCMs for treating IA was implemented to compare their efficacy and safety. Additionally, we considered the impact of treatment duration on efficacy and safety. Strict inclusion and exclusion criteria were applied. Only full-text RCTs were included, which were published in high-ranking journals and provided high-quality evidence.

However, there are also several limitations. The number of included studies in this analysis was relatively small. There were differences in standards of efficacy evaluation, characteristics of the patients, sample sizes, and outcome measures. The inadequate reporting of random sequence generation and allocation concealment in some included studies may introduce selection bias and performance bias. Inadequate allocation concealment increases the risk of selection bias, while the absence of blinding may introduce performance and detection biases. Consequently, there is a high probability of overestimating the efficacy of subjective outcomes, such as the clinical efficacy rate. Although the definition of the overall effective rate was explicitly established in this study, the metric remains highly subjective and is susceptible to performance bias and detection bias. The lack of blinding in the majority of the included studies further amplifies this risk of bias. This methodological limitation may introduce subjective tendencies from both investigators and participants during efficacy assessment, thereby causing the evaluated overall effective rate to deviate from the true therapeutic effect. Consequently, the limitations of this composite outcome are explicitly acknowledged, and the conclusions should be interpreted with caution. These methodological limitations may compromise the reliability of both the pooled effect sizes and the intervention rankings in this SNMA, ultimately downgrading the overall certainty of the evidence. Furthermore, Egger’s test, Begg’s test, and funnel plot analyses indicate the presence of publication bias in this study. This observation may be attributable to the fact that clinical trials with positive findings are more likely to be published. This bias influences our findings in two primary respects. First, it may lead to an overestimation of the pooled effect sizes. Given that studies with negative or neutral results were underrepresented, the pooled estimates could exaggerate the true clinical efficacy of CCPPs for IA. Second, it undermines the reliability of the treatment rankings by exaggerating the comparative advantages of certain interventions, which could potentially mislead clinical decision-making. Furthermore, given that young children with IA have a limited capacity to articulate their symptoms, outcome assessments are inherently subjective. These biases may exacerbate the deviations arising from such subjectivity, potentially leading to discrepancies between study findings and real-world clinical practice. This may compromise the validity of the conclusions regarding the effectiveness and safety of CCPPs. Some outcome measures in this analysis had fewer included studies, which could affect the results. There was also some publication bias. Notably, because the interventions evaluated in this study are CCPPs, their clinical application, dosing regimens, and indication assessments are fundamentally based on the physiological characteristics of Chinese children, the syndrome differentiation system of Traditional Chinese Medicine, and current pediatric clinical practice in China. Consequently, all included RCTs were conducted in China, and the study populations consisted entirely of Chinese infants and young children. These methodological and demographic characteristics indicate that our conclusions are primarily applicable to the management of IA within Chinese clinical settings. The generalizability of these findings may be limited, and they may not be directly applicable to pediatric populations from other countries, of different ethnic backgrounds, or with significantly different dietary and feeding practices. The conclusions concerning CCPP interventions for IA should be interpreted with caution regarding their application to pediatric pharmacotherapy in international settings. Therefore, it is necessary to apply more large-scale, multi-center, high-quality RCTs to provide more evidence.

## Data Availability

The original contributions presented in the study are included in the article/Supplementary Material, further inquiries can be directed to the corresponding author.
