# Supplementary material for: The effectiveness and safety of commercial Chinese polyherbal preparation in treating infantile anorexia: a systematic review and Bayesian network meta-analysis
Source: Front Pharmacol. 2026 Jun 18;17:1775817. doi: 10.3389/fphar.2026.1775817 (PMC13322854; doi:10.3389/fphar.2026.1775817)
Supplement: Supplementary file 1 [file Supplementaryfile1.doc]

**Table S1** Basic features of included studies

| First Author and Publication Year of the Included Studies | Intervention | n/Case | | Treatment Duration/d | Outcome Measure |
| --- | --- | --- | --- | --- | --- |
|  |  | T | C |  |  |
| L Yang [1] 2013 | SLBZKL+RD | 30 | 30 | 7 | ① |
| M Zou [2] 2014 | SLBZKL+RD | 36 | 36 | 28 | ① |
| XH Sha [3] 2016 | SLBZKL+RD | 90 | 90 | 28 | ① |
| HW Wang [4] 2015 | XEXJW+RD | 60 | 60 | 28 | ①② |
| X Jia [5] 1995 | AMLKFY | 100 | 51 | 30 | ①③ |
| JL Shao [6] 2015 | BLJPKL | 41 | 41 | 28 | ① |
| XX Li [7] 2011 | BLJPKL | 36 | 35 | 30 | ① |
| ZW Zhong [8] 2007 | BLJPKL | 54 | 37 | 30 | ① |
| SL Ma [9] 2010 | BLJPKL | 45 | 43 | 14 | ① |
| JJ Xia [10] 2013 | BEAKL | 60 | 60 | 15 | ① |
| YX Wen [11] 1998 | BZYQW | 50 | 50 | 90 | ① |
| HC Zhang [12] 2006 | BZYQW | 50 | 50 | 90 | ① |
| Y Wang [13] 2019 | EBKL+RD | 67 | 67 | 28 | ①③④ |
| LL Shi [14] 2023 | EBKL+RD | 49 | 49 | 28 | ①②③④ |
| LS Wan [15] 2002 | EBKL | 30 | 11 | 28 | ①③ |
| L Zhang [16] 2011 | EBKL | 51 | 51 | 28 | ① |
| X Wang [17] 2003 | EBKL | 47 | 41 | 20 | ① |
| WZ Quan [18] 2004 | EBKL | 60 | 40 | 45 | ① |
| YH Yang [19] 1998 | EKN | 40 | 36 | 20 | ① |
| XM Liu [20] 2022 | EPXKL | 100 | 100 | 21 | ①②④ |
| GJ Yin [21] 2017 | FEGJKL | 60 | 60 | - | ① |
| YH Yue [22] 2023 | FFTZSKL | 160 | 150 | 14 | ①④ |
| Y Zhang [23] 2016 | XEKKL | 45 | 45 | - | ① |
| L Liang [24] 2014 | HWLGKL | 35 | 35 | 28 | ① |
| JH Wu [25] 2007 | HJKFY | 56 | 56 | 28 | ①④ |
| XW Hu [26] 2007 | HQJKFY | 35 | 20 | 30 | ①④ |
| YC Li [27] 2016 | JBLKL | 43 | 43 | 42 | ① |
| J Li [28] 2016 | JBLKL | 62 | 61 | 56 | ①②③④ |
| HZ Tao [29] 2011 | JPW+RD | 51 | 48 | 14 | ① |
| YZ Kuang [30] 2004 | JPW | 66 | 42 | 42 | ① |
| LL Chen [31] 2016 | JPW | 65 | 65 | 28 | ① |
| SH Jiang [32] 2017 | JPW | 40 | 40 | 30 | ① |
| YQ Shen [33] 2011 | JWXSKFY | 100 | 52 | 15 | ① |
| WG Huang [34] 2013 | JWXSKFY | 60 | 58 | 14 | ① |
| YH Tao [35] 2016 | JWXSKFY+RD | 66 | 60 | 90 | ①③ |
| SY Hu [36] 2003 | JJKWKL | 121 | 122 | 14 | ①③④ |
| YH Li [37] 2016 | XEKWZSKL | 43 | 43 | 14 | ①④ |
| Q Chen [38] 2010 | XEKWZSKL+RD | 116 | 116 | 14 | ① |
| ZA Zhang [39] 2015 | KELKL | 92 | 34 | 28 | ① |
| Y Cao [40] 2005 | PKX | 54 | 37 | 28 | ① |
| SX Liu [41] 2012 | QHZKL | 100 | 80 | 21 | ① |
| SF Chen [42] 2015 | QZKFY | 60 | 60 | 14 | ① |
| L Li [43] 2021 | QZKFY | 36 | 36 | 28 | ①④ |
| YY Jin [44] 2018 | QZKFY | 30 | 30 | 28 | ①④ |
| GZ Zhu [45] 2003 | QPKFY | 45 | 40 | 14 | ①④ |
| GY Zhang [46] 2016 | QPW | 59 | 46 | 60 | ① |
| L Huang [47] 2011 | SJGRJ | 80 | 80 | 28 | ①④ |
| WH Wang [48] 2008 | SJGRJ | 86 | 78 | 21 | ① |
| XR Luo [49] 2014 | SBXSHJ+RD | 76 | 76 | 42 | ① |
| L Bu [50] 2006 | SMJPKFY | 40 | 40 | 15 | ① |
| SY Xu [51] 2005 | SMJPKFY | 64 | 56 | 14 | ① |
| HM Li [52] 2006 | SMJPKFY | 50 | 50 | 30 | ① |
| QF Huang [53] 2021 | SQXSKFY | 75 | 75 | 14 | ① |
| L Tang [54] 2019 | SQXSKFY+RD | 46 | 46 | 14 | ①④ |
| EC Chen [55] 2022 | SQXSKFY+RD | 30 | 30 | 28 | ①②③④ |
| J Liang [56] 2022 | SQXSKFY+RD | 50 | 50 | 14 | ① |
| XY Wu [57] 2017 | SQXSKFY+RD | 64 | 64 | 28 | ①②③ |
| X Chen [58] 2022 | SQXSKFY+RD | 58 | 58 | 28 | ①②③④ |
| XR Shi [59] 2020 | WSBCW+RD | 52 | 52 | 14 | ①③ |
| DL Li [60] 2013 | WSBCW | 21 | 21 | - | ①④ |
| Z Li [61] 2018 | WSBCW | 150 | 120 | - | ① |
| L Liu [62] 2008 | WSBCW+RD | 35 | 35 | 30 | ①② |
| ML Chen [63] 2000 | WSBCW | 86 | 37 | 30 | ① |
| YQ Zhang [64] 1999 | WSBCW | 120 | 90 | - | ① |
| LC Zhang [65] 2003 | SMTKFY | 60 | 40 | 14 | ① |
| JH Ji [66] 1998 | XSYWW | 36 | 36 | - | ① |
| MP Lin [67] 2004 | XECWKKL | 60 | 40 | 14 | ① |
| XR Bao [68] 2005 | XECWKKL | 120 | 80 | 14 | ① |
| JY Duan [69]2016 | XEFPKL+RD | 30 | 30 | 14 | ①④ |
| CP Li [70] 2015 | XEFPKL | 140 | 120 | 30 | ① |
| HG Jin [71] 2019 | XEFFJNJJJP+RD | 37 | 37 | 49 | ①②④ |
| Y Wang [72] 2021 | XEFFJNJJJP+RD | 118 | 117 | 21 | ①②④ |
| YW Sun [73] 2019 | XEJPKWHJ+RD | 40 | 40 | 30 | ①③④ |
| ZK Hong [74] 2020 | XEJPKWHJ+RD | 50 | 50 | 30 | ①③ |
| C Jiang [75] 2018 | XEJPW | 42 | 42 | 28 | ① |
| B Liu[76]2016 | XEJPW | 58 | 58 | 28 | ① |
| XY Zhang[77]2015 | XEJPW | 34 | 34 | 28 | ① |
| CY Hou[78]2012 | XEJPW | 70 | 70 | 28 | ① |
| ZH Yu[79]2011 | XEJPW | 53 | 53 | 21 | ① |
| HC Zhang[80]2016 | XEJWXSKFY+RD | 87 | 86 | 14 | ① |
| MZ Zhang[81]2020 | XEKKL+RD | 30 | 30 | 28 | ① |
| LR Wang[82]2010 | XEKKL | 65 | 50 | 14 | ① |
| WN Jia[83]2011 | XEKKL | 48 | 48 | 14 | ① |
| AL Zhao [84] 2012 | XEKKL | 80 | 55 | 28 | ① |
| HM Yan [85] 2008 | XEKKL | 90 | 30 | 14 | ①④ |
| YJ Zhao [86] 2012 | XEPWLKL | 70 | 67 | 28 | ①④ |
| YY Wang [87] 2017 | XEPWLKL | 63 | 63 | 28 | ①④ |
| ZM Yang [88] 2014 | XEPWLKL | 36 | 36 | 28 | ①②③④ |
| LM Lin [89] 2012 | XEPWLKL | 18 | 17 | 28 | ①②③ |
| SX Guo [90] 2020 | XEQZKFY | 113 | 111 | 28 | ①②③④ |
| CJ Huo [91] 2002 | XEWBW | 90 | 88 | 7 | ① |
| HM Zhang [92] 2012 | XEXISKL | 60 | 60 | 28 | ① |
| FL Li [93] 2009 | XEXISKL | 90 | 82 | 21 | ① |
| HG Kong [94] 2016 | XEXSKL | 53 | 53 | 28 | ① |
| XS Lin [95] 2012 | XEXSKL+RD | 60 | 60 | 28 | ① |
| BW Wu [96] 2018 | XEXSKL+RD | 90 | 90 | 28 | ①②④ |
| Y Bai [97] 2024 | XEXSKL+RD | 30 | 30 | 28 | ①③④ |
| M Lu[98] 2023 | XEXSKL+RD | 134 | 134 | 28 | ①④ |
| JH Ding [99] 2019 | XEYSKL | 40 | 40 | 14 | ① |
| XX Fang [100] 2019 | XEYSKL+RD | 80 | 160 | 56 | ①②④ |
| CH Zou [101] 2016 | XEYSKL+RD | 40 | 38 | 28 | ①②④ |
| HY Yu [102] 2017 | XEYSKL | 42 | 42 | - | ①② |
| QY Chen [103] 2016 | XPYEKL+RD | 40 | 40 | - | ①④ |
| GM Yu [104] 2016 | XPYEKL+RD | 80 | 160 | 28 | ① |
| JY Zhang [105] 2014 | XPYEKL | 40 | 40 | 28 | ①④ |
| L Cheng [106] 2017 | XPYEKL+RD | 43 | 43 | 30 | ①④ |
| YM Guo [107] 2021 | XPYEKL+RD | 50 | 50 | 28 | ①③ |
| XX Chen [108] 2015 | XPYEKL | 58 | 52 | 28 | ①④ |
| LH Wei [109] 2015 | XPYEKL+RD | 55 | 53 | 28 | ①④ |
| Y Zhang [110] 2011 | XPYEKL+RD | 64 | 64 | 28 | ①④ |
| Y Shen [111] 2022 | XPYEKL+RD | 30 | 30 | 14 | ① |
| P Wang [112] 2022 | XPYEKL+RD | 56 | 56 | 90 | ①②④ |
| KX Li [113]2019 | XPYEKL+RD | 33 | 33 | 56 | ①② |
| F Geng [114] 2020 | XPYEKL+RD | 62 | 61 | 15 | ①④ |
| D Huang [115] 2019 | XPYEKL+RD | 98 | 98 | 60 | ①②③ |
| QY Wang [116] 2018 | XPYEKL | 140 | 140 | 28 | ① |
| Y Qin [117] 2013 | XPYEKL+RD | 63 | 63 | - | ①④ |
| JH Fan [118] 2020 | XPYEKL+RD | 100 | 100 | 28 | ① |
| XP Liu [119]2011 | XPYEKL | 80 | 76 | 28 | ①④ |
| LS Zhou [120] 2013 | XPYEKL | 36 | 36 | 15 | ①④ |
| ML Wang [121] 2017 | XPYEKL | 43 | 43 | 60 | ①②④ |
| L He [122] 2009 | XPYEKL | 110 | 80 | 21 | ① |
| XM Huang [123] 2015 | XPYEKL | 25 | 25 | 28 | ①④ |
| H Hu [124] 2016 | XPYEKL | 65 | 65 | 56 | ① |
| GX Li [125] 2017 | XPYEKL+RD | 30 | 30 | 30 | ① |
| M Sun [126] 2020 | XPYEKL+RD | 46 | 46 | 30 | ①②④ |
| J Wan [127] 2018 | XPYEKL | 50 | 50 | 30 | ① |
| CX Ma [128] 2017 | XPYEKL+RD | 30 | 30 | 56 | ① |
| LL Chen [129] 2019 | XPYEKL+RD | 43 | 43 | 30 | ①②③ |
| YL Cheng [130] 2019 | XPYEKL | 43 | 43 | 56 | ① |
| YJ Fu [131] 2021 | XPYEKL+RD | 35 | 35 | 60 | ① |
| H Zhang [132] 2018 | XPYEKL | 50 | 50 | 30 | ① |
| JF Shi[133]2018 | XPYEKL | 46 | 46 | 28 | ① |
| HL Yin[134]2016 | XPYEKL | 86 | 78 | 21 | ① |
| J Lei[135]2021 | XPYEKL+RD | 75 | 75 | 14 | ① |
| YZ Qan[136]2022 | YSKKL | 200 | 100 | 14 | ①④ |
| AH Hu[137]2014 | XEYSKL | 42 | 39 | 28 | ①②④ |
| FF Lv[138]2015 | ZELKFY | 50 | 50 | 28 | ① |
| WT Zhang [139] 2015 | XEFPKL+RD | 92 | 95 | 14 | ①④ |
| F Huang [140] 2017 | XEKKL | 45 | 45 | 28 | ① |
| ZY Yang [141] 2022 | XEXSKL+RD | 44 | 44 | 30 | ①④ |

T: Trial group; C: Control group; AMLKFY: Anmole Koufuye (Anmole Oral Solution); BEAKL: Baoer’an Keli (Baoer’an Granules); BLJPKL: Bailing Jianpi Keli (Bailing Jianpi Granules); BZYQW: Buzhong Yiqi Wan(Buzhong Yiqi Pills); EBKL: Erbao Keli (Erbao Granules); EKN: Erkangning; EPXKL: Erpixing Keli (Erpixing Granules); FEGJKL: Feier Ganji Keli (Feier Ganji Granules); FFTZSKL: Fufang Taizishen Keli (Fufang Taizishen Granules); HJKFY: Huaji Koufuye (Huaji Oral Solution); HQJKFY: Huangqijing Koufuye (Huangqijing Oral Solution); HWLGKL: Hewei Liaogan Keli (Hewei Liaogan Granules); JBLKL: Jianbaoling Keli (Jianbaoling Granules); JJKWKL: Jinju Kaiwei Keli (Jinju Kaiwei Granules); JPW: Jianpi Wan (Jianpi Pills); JWXSKFY: Jianwei Xiaoshi Koufuye (Jianwei Xiaoshi Oral Solution); KELKL: Kangerling Keli (Kangerling Granules); PKX: Pikexin; QHZKL: Qihuozha Keli (Qihuozha Granules); QPKFY: Qipi Koufuye (Qipi Oral Solution); QPW: Qipi Wan (Qipi Pills); QZKFY: Qizao Koufuye (Qizao Oral Solution); SBXSHJ: Shanbai Xiaoshi Heji (Shanbai Xiaoshi Mixture); SJGRJ: Shaji Ganruji (Shaji Dry Emulsion); SLBZKL: Shenling Baizhu Keli (Shenling Baizhu Granules); SMJPKFY: Shanmai Jianpi Koufuye (Shanmai Jianpi Oral Solution); SMTKFY: Simo Tang Koufuye (Simo Decoction Oral Solution); SQXSKFY: Shenqu Xiaoshi Koufuye (Shenqu Xiaoshi Oral Solution); WSBCW: Wangshi Baochi Wan (Wangshi Baochi Pills); XECWKKL: Xiaoer Changweikang Keli (Xiaoer Changweikang Granules); XEFFJNJJJP: Xiaoer Fufang Jineijin Jupian (Xiaoer Fufang Jineijin Chewable Tablets); XEFPKL: Xiaoer Fupi Keli (Xiaoer Fupi Granules); XEJPKWHJ: Xiaoer Jianpi Kaiwei Heji (Xiaoer Jianpi Kaiwei Mixture); XEJPW: Xiaoer Jianpi Wan (Xiaoer Jianpi Pills); XEJWXSKFY: Xiaoer Jianwei Xiaoshi Koufuye ( Xiaoer Jianwei Xiaoshi Oral Solution); XEKKL: Xiaoer Kang Keli (Xiaoer Kang Granules); XEKWZSKL: Xiaoer Kaiwei Zengshi Keli (Xiaoer Kaiwei Zengshi Granules); XEPWLKL: Xiaoer Piweile Keli (Xiaoer Piweile Granules); XEQZKFY: Xiaoer Qizha Koufuye (Xiaoer Qizha Oral Solution); XEWBW: Xiaoer Weibao Wan (Xiaoer Weibao Pills); XEXISKL:Xiaoer Xishi Keli (Xiaoer Xishi Granules); XEXJW: Xiaoer Xiangju Wan (Xiaoer Xiangju Pills); XEXSKL: Xiaoer Xiaoshi Keli (Xiaoer Xiaoshi Granules); XEYSKFY: Xiaoer Yanshi Koufuye (Xiaoer Yanshi Oral Solution); XEYSKL: Xiaoer Yanshi Keli (Xiaoer Yanshi Granules); XPYEKL: Xingpi Yang’er Keli (Xingpi Yang’er Granules); XSYWW: Xiangsha Yangwei Wan (Xiangsha Yangwei Pills); YSKKL: Yanshi Kang Keli (Yanshi Kang Granules); ZELKFY: Zhangerling Koufuye (Zhangerling Oral Solution); ① Overall clinical effective rate; ② Changes in weight; ③ Level of hemoglobin; ④ Adverse reaction

**Reference**

1. Yang L, Zhang W, Quan Y, Zhu LL. Clinical Observation on Integrated Traditional Chinese and Western Medicine Therapy for Anorexia in Children. Shanxi Journal of Traditional Chinese Medicine. 2013;29(4):25. doi: 10.3969/j.issn.1000-7156.2013.04.013.

2. Zou M, Zu M, Li L. Efficacy Observation of Integrated Traditional Chinese and Western Medicine in Treating Anorexia in Children. Jiangxi Medical Journal. 2014;(7):635-6. doi: 10.3969/j.issn.1006-2238.2014.07.035.

3. Sha XH, Ma Y. Observation on the Efficacy of Integrated Traditional Chinese and Western Medicine in Treating Anorexia in Children. Cardiovascular Disease Journal of Integrated Traditional Chinese and Western Medicine (Electronic). 2016;4(19):2.

4. Wang HW, Feng B. Clinical Research on Integrated Traditional Chinese and Western Medicine for Anorexia. Henan Traditional Chinese Medicine. 2015;35(8):1968-70. doi: 10.16367/j.issn.1003-5028.2015.08.0826.

5. Jia X, Chen DG, Cheng XF. Clinical Application of Anmole Oral Solution for Infantile Anorexia. Strait Pharmaceutical Journal. 1995;(3):95.

6. Shao JL. Clinical Analysis of Bailing Jianpi Granules versus Zinc Preparations for Childhood Anorexia. Oriental Diet Therapy and Health Care. 2015;(8):220-.

7. Li XX. Clinical Study of Bailing Jianpi Granules for Infantile Anorexia. Chinese Community Doctors (Medical Specialty). 2011;13(31):180-1. doi: 10.3969/j.issn.1007-614x.2011.31.177.

8. Zhong ZW, Chen RR, Yan XR. Efficacy Analysis of Bailing Jianpi Granules for Infantile Anorexia. Zhejiang Journal of Integrated Traditional Chinese and Western Medicine. 2007;17(9):590. doi: 10.3969/j.issn.1005-4561.2007.09.048.

9. Ma SL. Observation on the Efficacy of Bailing Jianpi Granules for Infantile Anorexia. China Practical Medicine. 2010;5(31):143-4. doi: 10.3969/j.issn.1673-7555.2010.31.116.

10. Xia JJ. Bao'er'an Granules for 60 Cases of Infantile Anorexia. Medical Information. 2013;(11):674-.

11. Wen YX, Bao YQ. Treatment of 50 Cases of Infantile Anorexia with Buzhong Yiqi Pills. China's Naturopathy. 1998;(02):35. doi: 10.19621/j.cnki.11-3555/r.1998.02.046.

12. Zhang HC, Gao MF. Clinical Study on Buzhong Yiqi Pills for 50 Cases of Infantile Anorexia. Journal of Qiqihar Medical University. 2006;(09):1076-7.

13. Wang Y. Effect of Erbao Granules Combined with Conventional Therapy on Blood Trace Elements and Hemoglobin Levels in Children with Anorexia. Modern Diagnosis & Treatment. 2019;30(01):34-5.

14. Shi LL, Cui HM, Zhang JX, Zhang JF, Zhao LJ. Clinical Study of Erbao Granules Combined with Compound Digestive Enzyme Capsules for Infantile Anorexia. Drugs & Clinic. 2023;38(02):363-7.

15. Wan LS, Wang SC. Clinical Observation of Erbao Granules for Infantile Anorexia. Hunan Guiding Journal of Traditional Chinese Medicine and Pharmacology. 2002;(09):542-4. doi: 10.13862/j.cnki.cn43-1446/r.2002.09.019.

16. Zhang L. Clinical Analysis of 51 Cases of Infantile Anorexia Treated with Erbao Granules. Modern Journal of Integrated Traditional Chinese and Western Medicine. 2011;20(23):2901-2.

17. Wang X. Clinical Observation of Shibeigao Erbao Granules. Practical Clinical Journal of Integrated Traditional Chinese and Western Medicine. 2003;(06):24-5. doi: 10.13638/j.issn.1671-4040.2003.06.022.

18. Quan WZ. Treatment of 60 Cases of Childhood Anorexia with Shibeigao Erbao Granules. Shaanxi Journal of Traditional Chinese Medicine. 2004;(11):996.

19. Yang YH. Efficacy Observation of Erkangning for 40 Cases of Infantile Anorexia. Chinese Medical Journal of Metallurgical Industry. 1998;(06):31-2.

20. Liu XM, Wang XB. Clinical Efficacy and Safety of Epixing Granules for Anorexia in Children with Spleen Dysfunction Syndrome. Capital Medicine. 2022;(029-002).

21. Yin GJ. Efficacy Observation of Fei'er Ganji Granules for Childhood Anorexia. Strait Pharmaceutical Journal. 2017;29(03):186-7.

22. Yue YH, Yao L, Lai DC. Retrospective Study of Compound Taizishen Granules for Infantile Anorexia. Medical Innovation of China. 2023;20(26):143-7.

23. Zhang Y. Observation on Clinical Efficacy of Xiao'er Kang Granules for Infantile Anorexia. China Health Care & Nutrition. 2016;26(25):144-. doi: 10.3969/j.issn.1004-7484.2016.25.202.

24. Liang L. Efficacy Observation of Hewe Liaogan Granules for Infantile Anorexia. Clinical Journal of Chinese Medicine. 2014;26(12):1249-50. doi: 10.16448/j.cjtcm.2014.12.017.

25. Wu JH, Wang JZ. Treatment of 56 Cases of Infantile Anorexia with Huaji Oral Liquid. Jiangxi Journal of Traditional Chinese Medicine. 2007;(11):26-7.

26. Hu XW, Zhang M, Su SD. Efficacy Observation of Huangqi Jing Oral Liquid for 35 Cases of Infantile Anorexia. Medical Journal of National Defending Forces in Southwest China. 2007;(02):70.

27. Li YC, Li XQ. Efficacy of Jianbaoling Granules on Children with Anorexia and Its Effects on Blood Trace Elements and Immunity. Medical Journal of Chinese People's Health. 2016;28(09):57-8.

28. Li J, Zhang CW. Clinical Observation of Jianbaoling Granules for 62 Cases of Infantile Anorexia due to Spleen-Stomach Disharmony. Journal of Pediatrics of Traditional Chinese Medicine. 2016;12(04):50-2. doi: 10.16840/j.issn1673-4297.2016.04.17.

29. Tao HZ. Experience with Jianpi Pills plus Zinc Gluconate for Infantile Anorexia. Medical Information. 2011;24(09):4772-3.

30. Kuang YZ. Efficacy Observation of Jianpi Pills for 66 Cases of Infantile Anorexia. New Chinese Medicine. 2004;(04):26-7. doi: 10.13457/j.cnki.jncm.2004.04.013.

31. Chen LL. Clinical Efficacy Analysis of Jianpi Pills for Infantile Anorexia. Guangming Journal of Chinese Medicine. 2016;31(13):1851-2.

32. Jiang SH. Clinical Study of Jianpi Pills for Infantile Anorexia. Medical Forum. 2017;21(31):4385-6. doi: 10.19435/j.1672-1721.2017.31.083.

33. Shen YQ, Wang MJ. Efficacy Observation of Jianwei Xiaoshi Oral Liquid for Childhood Anorexia. Heilongjiang Medicine Journal. 2011;24(03):436-7. doi: 10.14035/j.cnki.hljyy.2011.03.015.

34. Huang WG, Tian XM. Efficacy Observation of Jianwei Xiaoshi Oral Liquid for Infantile Anorexia. Journal of Clinical Rational Drug Use. 2013;6(04):50. doi: 10.15887/j.cnki.13-1389/r.2013.04.010.

35. Tao YH. Clinical Observation of Jianwei Xiaoshi Oral Liquid for 66 Cases of Infantile Anorexia with Spleen-Stomach Deficiency. Journal of Pediatrics of Traditional Chinese Medicine. 2016;12(03):43-6. doi: 10.16840/j.issn1673-4297.2016.03.17.

36. Hu SY, Ma R, Liu HP. Clinical Study of Jinju Kaiwei Granules for Anorexia in Children with Spleen Dysfunction Syndrome. Chinese Journal of Information on Traditional Chinese Medicine. 2003;(07):16-7+78.

37. Li YH. Treatment of 43 Cases of Infantile Anorexia with Spleen Dysfunction Using Kaiwei Zengshi Granules. Fujian Journal of Traditional Chinese Medicine. 2016;47(06):64-5. doi: 10.13260/j.cnki.jfjtcm.011271.

38. Chen Q, Ma R, Hu SY, Wei XW. Clinical Observation of Xiao'er Kaiwei Zengshi Granules for Anorexia in Children with Spleen Dysfunction Syndrome. Tianjin Journal of Traditional Chinese Medicine. 2010;27(05):367-8.

39. Zhang ZA, Chen YH, Ling K, Gao XL. Clinical Efficacy Study of Kang'erling Granules for Infantile Anorexia. Beijing Medical Journal. 2015;37(02):182-3. doi: 10.15932/j.0253-9713.2015.2.031.

40. Cao Y. Efficacy Observation of Pikexin for 54 Cases of Infantile Anorexia. Fujian Medical Journal. 2005;(03):93-4.

41. Liu SX. Clinical Observation of Qihu Cha Granules for 100 Cases of Infantile Anorexia. Guide of China Medicine. 2012;10(05):226-7. doi: 10.15912/j.cnki.gocm.2012.05.033.

42. Chen SF. Qizao Oral Liquid Combined with Massage for 60 Cases of Infantile Anorexia with Spleen-Stomach Deficiency. China Pharmaceuticals. 2015;24(22):179-80.

43. Li L, Yang LJ, Xiong LJ. Observation on Clinical Efficacy of Qizao Oral Liquid for Infantile Anorexia. Medical Forum. 2021;25(14):2038-40. doi: 10.19435/j.1672-1721.2021.14.061.

44. Jin YY. Clinical Observation of Qizao Oral Liquid for Children with Anorexia. Guangming Journal of Chinese Medicine. 2018;33(08):1126-8.

45. Zhu GZ. Treatment of 45 Cases of Infantile Anorexia with Qipi Oral Liquid. Guangdong Medical Journal. 2003;(01):85. doi: 10.13820/j.cnki.gdyx.2003.01.065.

46. Zhang GY, Zhang J. Clinical Observation on Qipi Pills plus Zinc Gluconate for Infantile Anorexia. Guide of China Medicine. 2016;14(13):186-7. doi: 10.15912/j.cnki.gocm.2016.13.151.

47. Huang L, Yang Q. Treatment of 80 Cases of Infantile Anorexia with Hippophae Rhamnoides Dry Emulsion. Chinese Medicine Modern Distance Education of China. 2011;9(21):128-9.

48. Wang WH. Clinical Efficacy Observation of Hippophae Rhamnoides Dry Emulsion for Infantile Anorexia. Modern Health Care: Medical Innovation Research. 2008;5(12):2.

49. Luo XR, Zhang Y, Cai J. Efficacy Observation of Shanbai Xiaoshi Mixture Combined with Five Vitamins and Lysine for Infantile Anorexia. Modern Preventive Medicine. 2014;41(02):237-9.

50. Bu L, Yin H. Treatment of 40 Cases of Infantile Anorexia with Shanmai Jianpi Oral Liquid. Journal of Community Medicine. 2006;(24):34.

51. Xu SY, Liu ZS, Mo C. Treatment of 64 Cases of Infantile Anorexia with Shanmai Jianpi Oral Liquid. Chinese Journal of Integrated Traditional and Western Medicine on Digestion. 2005;(06):408-9.

52. Li HM. Efficacy Analysis of Shanmai Jianpi Oral Liquid for Infantile Anorexia. China Medical Abstracts (Pediatrics). 2006;(02):84.

53. Huang QF, Ren M, Niu Y, Zhang LH, Zhao JH, Dai WQ, et al. Effect of Shenqu Xiaoshi Oral Liquid on Infantile Anorexia. Health Must-Read. 2021;(23):201,3.

54. Tang L, Zheng XP. Clinical Study of Shenqu Xiaoshi Oral Liquid Combined with Licorice Zinc Granules for Infantile Anorexia. Drugs & Clinic. 2019;34(11):3310-3.

55. Chen EC. Effect of Shenqu Xiaoshi Oral Liquid Combined with Zinc Gluconate Granules on Serum Trace Elements and Hemoglobin Levels in Children with Anorexia. Practical Clinical Journal of Integrated Traditional Chinese and Western Medicine. 2022;22(03):61-3+82. doi: 10.13638/j.issn.1671-4040.2022.03.018.

56. Liang J. Efficacy Observation of Shenqu Xiaoshi Oral Liquid Combined with Zinc Preparation for Infantile Anorexia. Aerospace Medicine. 2022;33(03):277-9.

57. Wu XY, Song YY, Cheng B. Efficacy Observation of Shenqu Xiaoshi Oral Liquid Combined with Five Vitamins and Lysine for Anorexia. Herald of Traditional Chinese Medicine. 2017;23(23):107-10. doi: 10.13862/j.cnki.cn43-1446/r.2017.23.033.

58. Chen X, Li L, Wang L. Effect of Shenqu Xiaoshi Oral Liquid Combined with Licorice Zinc on Food Intake, Body Weight, Hemoglobin and Gastrointestinal Hormone Levels in Children with Anorexia. West China Medical Journal. 2022;34(11):1669-72+78.

59. Shi XR. Application Observation of Wangshi Baochi Pills in Children with Anorexia due to Spleen Dysfunction. Xinjiang Journal of Traditional Chinese Medicine. 2020;38(05):27-8.

60. Li DL. Randomized Parallel Controlled Study of Wangshi Baochi Pills Combined with Multienzyme Tablets for Infantile Anorexia. Journal of Practical Traditional Chinese Internal Medicine. 2013;27(17):4-5.

61. Li Z, Ni JX. Efficacy Observation of Wangshi Baochi Pills for Infantile Anorexia (Spleen-Stomach Damp-Heat Type). Shanghai Medical & Pharmaceutical Journal. 2018;39(13):18-9.

62. Liu L. Treatment of 35 Cases of Infantile Anorexia with Wangshi Baochi Pills. Tianjin Pharmacy. 2008;(02):49-50.

63. Chen ML. Treatment of 86 Cases of Infantile Anorexia with Wangshi Baochi Pills. Beijing Journal of Traditional Chinese Medicine. 2000;(04):62. doi: 10.16025/j.1674-1307.2000.04.035.

64. Zhang YQ, Hu H, Wang YH. Efficacy Observation of Wangshi Baochi Pills for Infantile Anorexia. Shanghai Journal of Traditional Chinese Medicine. 1999;(11):30. doi: 10.16305/j.1007-1334.1999.11.014.

65. Zhang LC. Treatment of 60 Cases of Infantile Anorexia with Wuma Simo Tang Oral Liquid. Sichuan Journal of Traditional Chinese Medicine. 2003;(02):54.

66. Ji JH. Clinical Observation of Xiangsha Yangwei Pills for Urban Children with Anorexia. Journal of Guiyang University of Traditional Chinese Medicine. 1998;(03):36-7. doi: 10.16588/j.cnki.issn1002-1108.1998.03.025.

67. Lin MP, Zhang J. Treatment of Infantile Anorexia with Xiao'er Changweikang Granules. Henan Traditional Chinese Medicine. 2004;(12):47. doi: 10.16367/j.issn.1003-5028.2004.12.037.

68. Bao XR. Efficacy Analysis of Xiao'er Changweikang Granules for 120 Cases of Infantile Anorexia. Jilin Medical Journal. 2005;(12):1346.

69. Duan JY. Observation on Efficacy and Safety of Xiao'er Fupi Granules Combined with Zinc Sulfate for Infantile Anorexia. Henan Medical Research. 2016;25(08):1475-6.

70. Li CP, Huang JL. Efficacy Observation of Xiao'er Fupi Granules for Infantile Anorexia. Practical Journal of Medicine & Pharmacy. 2015;32(07):627-8. doi: 10.14172/j.cnki.issn1671-4008.2015.07.029.

71. Jin HG. Efficacy Analysis of Xiao'er Compound Jineijin Chewable Tablets Combined with Live Combined B. Subtilis and E. Faecium Granules for 74 Cases of Anorexia. Medical Theory and Practice. 2019;32(13):2085-7. doi: 10.19381/j.issn.1001-7585.2019.13.062.

72. Wang Y, Zhong DD, Ji XH, Cheng YB, Gao XD, Zheng YM, et al. Multicenter Randomized Double-Blind Controlled Study of Xiao'er Compound Jineijin Chewable Tablets for Infantile Anorexia (Spleen-Stomach Disharmony Syndrome). China Journal of Chinese Materia Medica. 2021;46(09):2298-303. doi: 10.19540/j.cnki.cjcmm.20201125.501.

73. Sun YW, Hu Q. Clinical Study of Xiao'er Jianpi Kaiwei Mixture Combined with Lysine and Vitamin B12 Granules for Infantile Anorexia. Drugs & Clinic. 2019;34(10):3022-5.

74. Hong ZK, He XF. Clinical Study of Xiao'er Jianpi Kaiwei Mixture Combined with Lysine and Vitamin B12 Granules for Infantile Anorexia. Electronic Journal of General Stomatology. 2020;7(10):60-2.

75. Jiang C. Clinical Analysis of Xiao'er Jianpi Pills for Infantile Anorexia. Inner Mongolia Journal of Traditional Chinese Medicine. 2018;37(04):24-5. doi: 10.16040/j.cnki.cn15-1101.2018.04.013.

76. Liu B. Clinical Efficacy Evaluation of Xiao'er Jianpi Pills for Infantile Anorexia. Health for All (First Ten Days). 2016;10(1):36-7.

77. Zhang XY. Clinical Efficacy Evaluation of Xiao'er Jianpi Pills for Infantile Anorexia. China Continuing Medical Education. 2015;7(13):196-7.

78. Hou CY. Observation on Clinical Effect of Xiao'er Jianpi Pills for Infantile Anorexia. China Information on Traditional Chinese Medicine. 2012;4(5):254.

79. Yu ZH. Observation on Clinical Effect of Xiao'er Jianpi Pills for Infantile Anorexia. Jilin Medical Journal. 2011;32(33):7069. doi: 10.3969/j.issn.1004-0412.2011.33.080.

80. Zhang HC. Efficacy Analysis of Xiao'er Jianwei Xiaoshi Oral Liquid Combined with Zinc Gluconate for Infantile Anorexia. Electronic Journal of Clinical Medical Literature. 2016;3(07):1339+42. doi: 10.16281/j.cnki.jocml.2016.07.112.

81. Zhang MZ, Nong XY, Li YL, Huang JJ. Efficacy of Xiao'er Kang Granules Combined with Pricking Sifeng Acupoint for Infantile Anorexia. China Journal of Pharmaceutical Economics. 2020;15(10):106-8.

82. Wang LR. Clinical Observation of Xiao'er Kang Granules for 65 Cases of Infantile Anorexia. Clinical Medicine & Practice. 2010;19(08):490-1.

83. Jia WN, Tian XP. Clinical Observation of Xiao'er Kang Granules for Infantile Anorexia. World Health Digest. 2011;8(46):248-9. doi: 10.3969/j.issn.1672-5085.2011.46.210.

84. Zhao AL. Clinical Study of Xiao'er Kang Granules for Infantile Anorexia. World Health Digest. 2012;9(19):452-. doi: 10.3969/j.issn.1672-5085.2012.19.450.

85. Yan HM, Shu J. Clinical Observation of Xiao'er Kang Granules for Gastrointestinal Dysfunction Primarily Manifested as Anorexia. China Journal of Chinese Materia Medica. 2008;(17):2180-1.

86. Zhao YJ, Ma R, Hu SY, Wei XW. Supplementary Clinical Trial of Xiao'er Piwei Le Granules (Originally Xiao'er Kaiwei Zengshi Granules). Chinese Archives of Traditional Chinese Medicine. 2012;30(07):1534-6. doi: 10.13193/j.archtcm.2012.07.96.zhaoyj.055.

87. Wang YY, Song QX, He YY, Liao F. Efficacy of Xiao'er Piwei Le Granules for Anorexia in Children with Spleen Dysfunction and Its Effect on Serum Leptin Levels. Journal of Yunnan University of Traditional Chinese Medicine. 2017;40(06):53-5. doi: 10.19288/j.cnki.issn.1000-2723.2017.06.015.

88. Yang ZM, Meng CL. Treatment of Infantile Anorexia with Xiao'er Piwei Le Granules. Journal of Changchun University of Chinese Medicine. 2014;30(05):927-9. doi: 10.13463/j.cnki.cczyy.2014.05.065.

89. Lin LM, Liu JZ, Xiang XX. Clinical Study of Xiao'er Piwei Le for Infantile Anorexia (Spleen Dysfunction Syndrome). Hubei Journal of Traditional Chinese Medicine. 2013;35(11):9-10.

90. Guo SX, Hu SY, Ma R, Zhong CL, Liu H, Liu XF, et al. Journal of Pediatrics of Traditional Chinese Medicine. Journal of Pediatrics of Traditional Chinese Medicine. 2020;16(01):37-42. doi: 10.16840/j.issn1673-4297.2020.01.12.

91. Huo CJ, Xu Y, Niu BT, Wang Y. Treatment of 90 Cases of Anorexia with Xiao'er Weibao Pills. Henan Medical Information. 2002;(07):42.

92. Zhang HM. Treatment of 60 Cases of Infantile Anorexia with Xiao'er Xishi Granules. Journal of Community Medicine. 2012;10(17):21-2.

93. Li FL, Li WJ, Li WQ. Efficacy Observation of Xiao'er Xishi Granules for 90 Cases of Infantile Anorexia. Journal of Pediatrics of Traditional Chinese Medicine. 2009;5(05):26-8.

94. Kong HG. Effect of Xiao'er Xiaoshi Granules on Gastrointestinal Motility and Gastrin Secretion in Children with Anorexia. New Chinese Medicine. 2016;48(07):192-3. doi: 10.13457/j.cnki.jncm.2016.07.083.

95. Lin XS, Ye J, Ding WN. Integrated Traditional Chinese and Western Medicine for 60 Cases of Infantile Anorexia. Zhejiang Journal of Traditional Chinese Medicine. 2012;47(11):806.

96. Wu BW, Li XY. Clinical Observation of Integrated Traditional Chinese and Western Medicine for 90 Cases of Infantile Anorexia. Chinese Journal of Ethnomedicine and Ethnopharmacy. 2018;27(4):77-8. doi: 10.3969/j.issn.1007-8517.2018.4.zgmzmjyyzz201804026.

97. Bai Y, Li HP, Li H, Zhang J. Effect and Mechanism of Xiao'er Xiaoshi Granules on Anorexia-Related Factors in Children. Chinese Archives of Traditional Chinese Medicine. 2024;42(02):75-9. doi: 10.13193/j.issn.1673-7717.2024.02.017.

98. Lu M, Zou SN. Clinical Study of Xiao'er Xiaoshi Granules Combined with Quadruple Viable Bacteria Tablets for Infantile Anorexia. New Chinese Medicine. 2023;55(01):125-8. doi: 10.13457/j.cnki.jncm.2023.01.027.

99. Ding JH. Effect of Xiao'er Yanshi Granules on Plasma Neuropeptide Y and Serum Leptin Levels in Children with Anorexia. Health Guide. 2019;(30):345.

100. Fang XX, Wang YC, Zhu LK, Yao JC, Hu GH. Clinical Efficacy of Xiao'er Yanshi Granules Combined with Saccharomyces Boulardii for Infantile Anorexia and Its Effect on Serum Cytokines. Chinese Journal of Experimental Traditional Medical Formulae. 2019;25(12):115-20. doi: 10.13422/j.cnki.syfjx.20191123.

101. Zou CH, Shen SZ. Clinical Observation of Xiao'er Yanshi Granules Combined with New Selenium Supplement for 40 Cases of Infantile Anorexia. Journal of Pediatrics of Traditional Chinese Medicine. 2016;12(03):49-52. doi: 10.16840/j.issn1673-4297.2016.03.19.

102. Yu HY. Efficacy of Xiao'er Yanshi Granules for Anorexia in Children and Its Effect on Serum Ghrelin and Leptin Levels. Modern Journal of Integrated Traditional Chinese and Western Medicine. 2017;26(08):857-9.

103. Chen QY. Clinical Efficacy Observation of Xingpi Yang'er Granules for Infantile Anorexia. China Practical Medicine. 2016;11(15):213-4. doi: 10.14163/j.cnki.11-5547/r.2016.15.157.

104. Yu GM. Clinical observation of two Chinese medicines combined for treating infantile anorexia. Chinese Journal of Urban and Rural Enterprise Hygiene. 2016;31(04):86-8. doi: 10.16286/j.1003-5052.2016.04.036.

105. Zhang JY, Lin K, Cao FE, Yang HH. Clinical observation of 40 cases of infantile anorexia treated with integrated traditional Chinese and Western medicine. Internal Medicine of China. 2014;9(06):690-1. doi: 10.16121/j.cnki.cn45-1347/r.2014.06.023.

106. Cheng L. Analysis of clinical efficacy of Xingpi Yang'er Granules in treating infantile anorexia. Contemporary Medicine. 2017;23(24):145-7.

107. Guo YM. Observation on the efficacy of Xingpi Yang'er Granules for infantile anorexia. Marriage & Health. 2021;(20):1-2.

108. Chen XX, Bu YQ. Effects of Xingpi Yang'er Granules on serum gastrin, plasma motilin, and neuropeptide Y levels in children with anorexia. Chinese Journal of Integrated Traditional and Western Medicine on Digestion. 2015;23(03):180-3.

109. Wei LH. Clinical study on adjuvant treatment of infantile anorexia with Xingpi Yang'er Granules. New Chinese Medicine. 2015;47(09):165-6. doi: 10.13457/j.cnki.jncm.2015.09.078.

110. Zhang Y, Chen P. Treatment of 64 cases of infantile anorexia with Xingpi Yang'er Granules combined with Golden Bifido. Chinese Medicine Modern Distance Education of China. 2011;9(20):37-8.

111. Shen Y, Kong Y. Analysis of clinical efficacy of Xingpi Yang'er Granules combined with Mamiai for infantile anorexia. Kangyi. 2022;(18):136-8. doi: 10.12332/j.issn.2095-6525.2022.18.046.

112. Wang P, Xue L. Clinical efficacy of Xingpi Yang'er Granules combined with Bifidobacterium and Lactobacillus Triple Viable Capsules and Zinc Gluconate Granules in treating infantile anorexia. Journal of Clinical Research. 2022;39(4):572-4,8. doi: 10.3969/j.issn.1671-7171.2022.04.026.

113. Li KX. Evaluation of the efficacy of Xingpi Yang'er Granules combined with Bifidobacterium Triple Viable and Zinc Gluconate Oral Solution in 33 cases of infantile anorexia. China Health Care & Nutrition. 2019;29(25):366-7.

114. Geng F. Clinical study on Xingpi Yang'er Granules combined with Bifidobacterium Triple Viable Capsules and zinc supplementation for infantile anorexia. Journal of Diseases Monitor & Control. 2020;14(5):361-3,72. doi: 10.19891/j.issn1673-9388.(2020)05-0361-04.

115. Huang D. Clinical study of Xingpi Yang'er Granules combined with Bifidobacterium Triple Viable Capsules and synchronous zinc supplementation in 98 cases of infantile anorexia. Journal of Medical Forum. 2019;40(2):143-4.

116. Wang QY, Zhong JH. Observation on clinical efficacy of Xingpi Yang'er Granules for infantile anorexia. China Health Care & Nutrition. 2018;28(15):178. doi: 10.3969/j.issn.1004-7484.2018.15.160.

117. Qin Y. Study on the application of Xingpi Yang'er Granules in treating infantile anorexia. Chinese and Foreign Medical Research. 2013;(4):39.

118. Fan JH. Xingpi Yang'er Granules for 200 cases of infantile anorexia. Electronic Journal of Clinical Medical Literature. 2020;7(10):24-5.

119. Liu XP, Ma YH, Liu J. Xingpi Yang'er Granules for 80 cases of infantile anorexia. Shaanxi Journal of Traditional Chinese Medicine. 2011;32(10):1331-2.

120. Zhou LS. Clinical analysis of Xingpi Yang'er Granules for infantile anorexia. China Health Care and Nutrition (Mid-month Issue). 2013;(10):520-1.

121. Wang ML, Wen Y. Clinical analysis of Xingpi Yang'er Granules for infantile anorexia. World Latest Medicine Information. 2017;17(98):97. doi: 10.19613/j.cnki.1671-3141.2017.98.083.

122. He L. Efficacy observation of Xingpi Yang'er Granules for 110 cases of infantile anorexia. Chinese Community Doctors. 2009;25(13):41.

123. Huang XM. Clinical observation of Xingpi Yang'er Granules for 50 cases of infantile anorexia. Journal of Frontiers of Medicine. 2015;5(27):150-1. doi: 10.3969/j.issn.2095-1752.2015.27.139.

124. Hu H, Chen F. Clinical observation of Xingpi Yang'er Granules for 65 cases of infantile anorexia. New Chinese Medicine. 2016;48(02):165-7. doi: 10.13457/j.cnki.jncm.2016.02.063.

125. Li GX. Clinical observation of Xingpi Yang'er Granules for infantile anorexia. Journal of Clinical Medical Literature. 2017;4(82):16137-8. doi: 10.16281/j.cnki.jocml.2017.82.055.

126. Sun M. Clinical observation of Xingpi Yang'er Granules for infantile anorexia. China Practical Medicine. 2020;15(05):153-5. doi: 10.14163/j.cnki.11-5547/r.2020.05.069.

127. Wan J. Clinical observation of Xingpi Yang'er Granules for infantile anorexia. Journal of Frontiers of Medicine. 2018;8(13):24-5. doi: 10.3969/j.issn.2095-1752.2018.13.017.

128. Ma CX. Observation on clinical efficacy of Xingpi Yang'er Granules for infantile anorexia. Journal of China Prescription Drug. 2017;15(11):107-8.

129. Chen LL, Xu XQ, Wang HH, Zhao NK, Zhang ZA. Journal of Practical Medical Techniques. Journal of Practical Medical Techniques. 2019;26(04):470-1. doi: 10.19522/j.cnki.1671-5098.2019.04.036.

130. Cheng YL. Efficacy observation of Xingpi Yang'er Granules for infantile anorexia. Northern Pharmacy 2019;16(07):76-7.

131. Fu YJ. Efficacy observation and effectiveness evaluation of Xingpi Yang'er Granules for infantile anorexia. Maternal & Child World. 2021;(31):141,2.

132. Zhang H. Evaluation of medication efficacy of Xingpi Yang'er Granules for infantile anorexia. Health for Everyone. 2018;(22):238.

133. Shi JF, Zhang FH. Efficacy observation of Xingpi Yang'er Granules for infantile anorexia. Capital Food Medicine. 2018;25(09):103.

134. Yin HL. Observation on clinical efficacy of Xingpi Yang'er Granules for infantile anorexia. Maternal & Child World. 2016;(4):85.

135. Lei J. Clinical observation on efficacy of Xingpi Yang'er Granules for infantile anorexia. Family Life Guide. 2021;37(24):165-6.

136. Qin YZ, Wang DY. Multicenter clinical study of Anshikang Granules for infantile anorexia with spleen dysfunction syndrome. World Journal of Integrated Traditional and Western Medicine. 2022;17(11):2211-4. doi: 10.13935/j.cnki.sjzx.221115.

137. Hu AH, Xu HM, Hu GH, Jin F, Li Z, Fang GX. Changes in peripheral blood appetite regulators in anorexic children and intervention effect of Xiao'er Anshi Granules. China Journal of Chinese Materia Medica. 2014;39(23):4685-8.

138. Lyu FF. Clinical efficacy observation of Zhuangerling Oral Liquid for infantile anorexia with spleen-stomach qi deficiency syndrome. Chinese Journal of Medical Guide. 2015;27(01):33-4.

139. Zhang WT, Zhang YB, Chen ZY, Chen CB, Zhao HM, You JY. Therapeutic effect of Xiao'er Fupi Granules combined with zinc sulfate on infantile anorexia. Chinese Journal of Physicians. 2015;17(12):1869-71. doi: 10.3760/cma.j.issn.1008-1372.2015.12.032.

140. Huang F, Zheng DL. Clinical study on Xiao'er Kang Granules for infantile anorexia. World Latest Medicine Information. 2017;17(14):112+4.

141. Yang ZY, Lin XC. Clinical efficacy of Xiao'er Xiaoshi Granules combined with Bifidobacterium Tetragenous Viable Tablets in treating infantile anorexia. Journal of New Chinese Medicine. 2022;54(22):106-9. doi: 10.13457/j.cnki.jncm.2022.22.024.

**Table S2** League table for the overall effective rate of commercial Chinese polyherbal preparations in treating infantile anorexia

| Interventions | RR[95% CI] | | | | | | | | | | |
| --- | --- | --- | --- | --- | --- | --- | --- | --- | --- | --- | --- |
|  | AMLKFY | BEAKL | BLJPKL | BZYQW | EBKL | EBKLandRD | EKN | EPXKL | FEGJKLandRD | FFTZSKL | RD |
| AMLKFY | 0 |  |  |  |  |  |  |  |  |  |  |
| BEAKL | 1.30 (0.94, 1.85) | 0 |  |  |  |  |  |  |  |  |  |
| BLJPKL | 1.18 (0.89, 1.63) | 0.91 (0.73, 1.13) | 0 |  |  |  |  |  |  |  |  |
| BZYQW | 1.08 (0.80, 1.52) | 0.83 (0.65, 1.07) | 0.92 (0.75, 1.12) | 0 |  |  |  |  |  |  |  |
| EBKL | 1.09 (0.81, 1.51) | 0.84 (0.67, 1.06) | 0.92 (0.77, 1.11) | 1.00 (0.82, 1.25) | 0 |  |  |  |  |  |  |
| EBKLandRD | 1.26 (0.94, 1.75) | 0.97 (0.77, 1.22) | 1.07 (0.89, 1.28) | 1.16 (0.94, 1.44) | 0.77 (0.51, 1.10) | 0 |  |  |  |  |  |
| EKN | 0.97 (0.60, 1.50) | 0.74 (0.48, 1.08) | 0.82 (0.54, 1.17) | 0.89 (0.58, 1.29) | 1.05 (0.84, 1.31) | 1.37 (0.94, 2.09) | 0 |  |  |  |  |
| EPXKL | 1.32 (0.97, 1.86) | 1.01 (0.79, 1.31) | 1.12 (0.90, 1.38) | 1.22 (0.96, 1.55) | 1.03 (0.82, 1.30) | 1.35 (0.92, 2.07) | 0.99 (0.76, 1.27) | 0 |  |  |  |
| FEGJKLandRD | 1.30 (0.94, 1.84) | 1.00 (0.77, 1.30) | 1.10 (0.88, 1.37) | 1.20 (0.94, 1.54) | 0.97 (0.78, 1.21) | 1.27 (0.88, 1.94) | 0.93 (0.73, 1.18) | 0.94 (0.74, 1.21) | 0 |  |  |
| FFTZSKL | 1.22 (0.90, 1.73) | 0.94 (0.74, 1.21) | 1.04 (0.85, 1.28) | 1.13 (0.90, 1.43) | 0.77 (0.51, 1.10) | 0.95 (0.61, 1.52) | 0.69 (0.49, 0.96) | 0.70 (0.50, 0.98) | 0.94 (0.74, 1.21) | 0 |  |
| RD | 1.48 (1.15, 2.00) | 1.14 (0.95, 1.38) | 1.26 (1.12, 1.42) | 1.37 (1.17, 1.62) | 1.36 (1.20, 1.57) | 1.18 (1.03, 1.36) | 1.54 (1.10, 2.27) | 1.12 (0.95, 1.34) | 1.14 (0.95, 1.38) | 1.21 (1.03, 1.43) | 0 |

**Table S3** League table for the overall effective rate of commercial Chinese polyherbal preparations in treating infantile anorexia

| Interventions | RR[95% CI] | | | | | | | | | |
| --- | --- | --- | --- | --- | --- | --- | --- | --- | --- | --- |
|  | HJKFY | HQJKFY | HWLGKL | JBLKL | JJKWKL | JPW | JPWandRD | JWXSKFY | JWXSKFYandRD | RD |
| HJKFY | 0 |  |  |  |  |  |  |  |  |  |
| HQJKFY | 1.34 (0.85, 2.05) | 0 |  |  |  |  |  |  |  |  |
| HWLGKL | 1.20 (0.78, 1.82) | 0.89 (0.57, 1.44) | 0 |  |  |  |  |  |  |  |
| JBLKL | 1.34 (0.98, 1.88) | 0.99 (0.71, 1.50) | 1.12 (0.80, 1.60) | 0 |  |  |  |  |  |  |
| JJKWKL | 1.39 (1.02, 1.94) | 1.03 (0.74, 1.55) | 1.16 (0.83, 1.66) | 1.04 (0.83, 1.30) | 0 |  |  |  |  |  |
| JPW | 1.31 (0.97, 1.81) | 0.97 (0.71, 1.45) | 1.09 (0.79, 1.54) | 0.98 (0.79, 1.20) | 0.94 (0.77, 1.15) | 0 |  |  |  |  |
| JPWandRD | 1.03 (0.71, 1.51) | 0.76 (0.51, 1.19) | 0.86 (0.58, 1.28) | 0.77 (0.56, 1.03) | 0.74 (0.55, 0.98) | 0.79 (0.61, 0.98) | 0 |  |  |  |
| JWXSKFY | 1.40 (1.03, 1.95) | 1.04 (0.75, 1.55) | 1.17 (0.85, 1.67) | 1.05 (0.84, 1.30) | 1.01 (0.82, 1.24) | 1.08 (0.89, 1.30) | 1.36 (1.04, 1.84) | 0 |  |  |
| JWXSKFYandRD | 1.22 (0.86, 1.75) | 0.91 (0.63, 1.38) | 1.02 (0.71, 1.49) | 0.91 (0.70, 1.18) | 0.88 (0.68, 1.13) | 0.94 (0.73, 1.18) | 1.19 (0.87, 1.65) | 0.87 (0.67, 1.11) | 0 |  |
| RD | 1.62 (1.24, 2.19) | 1.20 (0.90, 1.75) | 1.35 (1.01, 1.87) | 1.21 (1.03, 1.43) | 1.17 (1.01, 1.36) | 1.24 (1.09, 1.42) | 1.57 (1.25, 2.06) | 1.15 (1.01, 1.34) | 1.32 (1.09, 1.65) | 0 |

**Table S4** League table for the overall effective rate of commercial Chinese polyherbal preparations in treating infantile anorexia

| Interventions | RR[95% CI] | | | | | | | | | | |
| --- | --- | --- | --- | --- | --- | --- | --- | --- | --- | --- | --- |
|  | KELKL | PKX | QHZKL | QPKFY | QPWandRD | QZKFY | SBXSHJandRD | SJGRJ | SLBZKLandRD | SMJPKFY | RD |
| KELKL | 0 |  |  |  |  |  |  |  |  |  |  |
| PKX | 0.85 (0.58, 1.22) | 0 |  |  |  |  |  |  |  |  |  |
| QHZKL | 0.96 (0.73, 1.31) | 1.14 (0.82, 1.63) | 0 |  |  |  |  |  |  |  |  |
| QPKFY | 1.07 (0.80, 1.46) | 1.26 (0.90, 1.81) | 1.11 (0.85, 1.44) | 0 |  |  |  |  |  |  |  |
| QPWandRD | 0.90 (0.65, 1.27) | 1.07 (0.73, 1.58) | 0.94 (0.69, 1.26) | 0.85 (0.61, 1.15) | 0 |  |  |  |  |  |  |
| QZKFY | 0.94 (0.73, 1.25) | 1.11 (0.82, 1.57) | 0.98 (0.78, 1.23) | 0.88 (0.70, 1.12) | 1.04 (0.79, 1.40) | 0 |  |  |  |  |  |
| SBXSHJandRD | 0.86 (0.62, 1.20) | 1.01 (0.71, 1.49) | 0.89 (0.66, 1.19) | 0.80 (0.60, 1.08) | 0.95 (0.68, 1.34) | 0.91 (0.69, 1.18) | 0 |  |  |  |  |
| SJGRJ | 0.94 (0.73, 1.25) | 1.10 (0.81, 1.56) | 0.97 (0.77, 1.23) | 0.87 (0.69, 1.11) | 1.03 (0.79, 1.40) | 0.99 (0.81, 1.21) | 1.09 (0.84, 1.44) | 0 |  |  |  |
| SLBZKLandRD | 0.93 (0.73, 1.24) | 1.10 (0.81, 1.56) | 0.97 (0.78, 1.22) | 0.87 (0.70, 1.11) | 1.03 (0.79, 1.39) | 0.99 (0.82, 1.21) | 1.09 (0.84, 1.43) | 1.00 (0.82, 1.21) | 0 |  |  |
| SMJPKFY | 0.98 (0.77, 1.30) | 1.16 (0.86, 1.64) | 1.02 (0.82, 1.28) | 0.92 (0.74, 1.16) | 1.09 (0.83, 1.45) | 1.04 (0.87, 1.26) | 1.15 (0.89, 1.50) | 1.05 (0.87, 1.27) | 1.05 (0.87, 1.27) | 0 |  |
| RD | 1.20 (0.98, 1.54) | 1.41 (1.08, 1.95) | 1.24 (1.05, 1.50) | 1.12 (0.94, 1.37) | 1.32 (1.05, 1.73) | 1.27 (1.11, 1.47) | 1.40 (1.12, 1.78) | 1.28 (1.11, 1.48) | 1.28 (1.13, 1.47) | 1.22 (1.08, 1.39) | 0 |

**Table S5** League table for the overall effective rate of commercial Chinese polyherbal preparations in treating infantile anorexia

| Interventions | RR[95% CI] | | | | | | | | | | |
| --- | --- | --- | --- | --- | --- | --- | --- | --- | --- | --- | --- |
|  | SMTKFYandRD | SQXSKFY | SQXSKFYandRD | WSBCW | WSBCWandRD | XECWKKL | XEFFJNJJJP | XEFFJNJJJPandRD | XEFPKL | XEFPKLandRD | RD |
| SMTKFYandRD | 0 |  |  |  |  |  |  |  |  |  |  |
| SQXSKFY | 1.15 (0.89, 1.54) | 0 |  |  |  |  |  |  |  |  |  |
| SQXSKFYandRD | 1.07 (0.86, 1.38) | 0.93 (0.77, 1.12) | 0 |  |  |  |  |  |  |  |  |
| WSBCW | 1.17 (0.95, 1.51) | 1.02 (0.85, 1.23) | 1.10 (0.97, 1.24) | 0 |  |  |  |  |  |  |  |
| WSBCWandRD | 1.17 (0.90, 1.56) | 1.02 (0.80, 1.28) | 1.09 (0.90, 1.31) | 1.00 (0.82, 1.19) | 0 |  |  |  |  |  |  |
| XECWKKL | 1.12 (0.88, 1.47) | 0.97 (0.79, 1.20) | 1.05 (0.90, 1.22) | 0.95 (0.82, 1.11) | 0.96 (0.78, 1.19) | 0 |  |  |  |  |  |
| XEFFJNJJJP | 1.26 (0.98, 1.69) | 1.10 (0.87, 1.38) | 1.18 (0.98, 1.42) | 1.07 (0.89, 1.30) | 1.08 (0.86, 1.37) | 1.13 (0.92, 1.39) | 0 |  |  |  |  |
| XEFFJNJJJPandRD | 1.10 (0.81, 1.49) | 0.95 (0.72, 1.24) | 1.03 (0.80, 1.28) | 0.94 (0.73, 1.16) | 0.94 (0.71, 1.23) | 0.98 (0.76, 1.25) | 0.87 (0.66, 1.13) | 0 |  |  |  |
| XEFPKL | 0.91 (0.68, 1.25) | 0.79 (0.60, 1.04) | 0.85 (0.67, 1.07) | 0.78 (0.61, 0.97) | 0.78 (0.59, 1.02) | 0.81 (0.63, 1.04) | 0.72 (0.55, 0.94) | 0.83 (0.62, 1.13) | 0 |  |  |
| XEFPKLandRD | 1.06 (0.83, 1.39) | 0.92 (0.74, 1.14) | 0.99 (0.84, 1.16) | 0.90 (0.76, 1.06) | 0.90 (0.73, 1.13) | 0.94 (0.78, 1.14) | 0.84 (0.67, 1.04) | 0.96 (0.75, 1.26) | 1.16 (0.90, 1.50) | 0 |  |
| RD | 1.27 (1.05, 1.62) | 1.11 (0.94, 1.31) | 1.19 (1.10, 1.31) | 1.09 (1.00, 1.19) | 1.09 (0.93, 1.30) | 1.14 (1.01, 1.30) | 1.01 (0.86, 1.19) | 1.16 (0.95, 1.46) | 1.40 (1.14, 1.75) | 1.21 (1.05, 1.39) | 0 |

**Table S6** League table for the overall effective rate of commercial Chinese polyherbal preparations in treating infantile anorexia

| Interventions | RR[95% CI] | | | | | | | | | | |
| --- | --- | --- | --- | --- | --- | --- | --- | --- | --- | --- | --- |
|  | XEJPKWHJandRD | XEJPW | XEJWXSKFYandRD | XEKKL | XEKKLandRD | XEKWZSKL | XEPWLKL | XEQZKFY | XEWBW | XEXISKL | RD |
| XEJPKWHJandRD | 0 |  |  |  |  |  |  |  |  |  |  |
| XEJPW | 0.95 (0.80, 1.13) | 0 |  |  |  |  |  |  |  |  |  |
| XEJWXSKFYandRD | 0.76 (0.56, 1.02) | 0.81 (0.60, 1.06) | 0 |  |  |  |  |  |  |  |  |
| XEKKL | 1.01 (0.86, 1.20) | 1.07 (0.94, 1.22) | 1.33 (1.02, 1.76) | 0 |  |  |  |  |  |  |  |
| XEKKLandRD | 0.97 (0.72, 1.28) | 1.03 (0.77, 1.32) | 1.27 (0.88, 1.82) | 0.96 (0.72, 1.23) | 0 |  |  |  |  |  |  |
| XEKWZSKL | 1.15 (0.90, 1.48) | 1.22 (0.98, 1.52) | 1.51 (1.10, 2.12) | 1.14 (0.92, 1.41) | 1.19 (0.88, 1.66) | 0 |  |  |  |  |  |
| XEPWLKL | 0.79 (0.64, 0.96) | 0.83 (0.70, 0.99) | 1.03 (0.77, 1.40) | 0.78 (0.66, 0.91) | 0.81 (0.62, 1.09) | 0.68 (0.53, 0.87) | 0 |  |  |  |  |
| XEQZKFY | 1.13 (0.93, 1.38) | 1.19 (1.01, 1.42) | 1.48 (1.11, 2.01) | 1.12 (0.95, 1.31) | 1.16 (0.89, 1.58) | 0.98 (0.77, 1.25) | 1.44 (1.18, 1.76) | 0 |  |  |  |
| XEWBW | 0.97 (0.77, 1.22) | 1.02 (0.83, 1.26) | 1.27 (0.93, 1.76) | 0.96 (0.78, 1.16) | 1.00 (0.74, 1.38) | 0.84 (0.64, 1.10) | 1.23 (0.98, 1.55) | 0.86 (0.68, 1.07) | 0 |  |  |
| XEXISKL | 0.92 (0.76, 1.13) | 0.97 (0.82, 1.15) | 1.21 (0.91, 1.64) | 0.91 (0.78, 1.07) | 0.95 (0.73, 1.29) | 0.80 (0.63, 1.01) | 1.17 (0.97, 1.43) | 0.82 (0.67, 0.99) | 0.95 (0.76, 1.20) | 0 |  |
| RD | 1.17 (1.02, 1.37) | 1.24 (1.12, 1.38) | 1.54 (1.20, 2.02) | 1.16 (1.07, 1.26) | 1.21 (0.96, 1.59) | 1.02 (0.84, 1.24) | 1.49 (1.30, 1.73) | 1.04 (0.91, 1.20) | 1.21 (1.02, 1.46) | 1.27 (1.12, 1.47) | 0 |

**Table S7** League table for the overall effective rate of commercial Chinese polyherbal preparations in treating infantile anorexia

| Interventions | RR[95% CI] | | | | | | | | | | |
| --- | --- | --- | --- | --- | --- | --- | --- | --- | --- | --- | --- |
|  | XEXJWandRD | XEXSKL | XEXSKLandRD | XEYSKL | XEYSKLandRD | XPYEKL | XPYEKLandRD | XSYWW | YSKKL | ZELKFY | RD |
| XEXJWandRD | 0 |  |  |  |  |  |  |  |  |  |  |
| XEXSKL | 1.00 (0.77, 1.30) | 0 |  |  |  |  |  |  |  |  |  |
| XEXSKLandRD | 1.00 (0.83, 1.23) | 1.00 (0.82, 1.24) | 0 |  |  |  |  |  |  |  |  |
| XEYSKL | 0.98 (0.79, 1.21) | 0.98 (0.79, 1.22) | 0.97 (0.85, 1.12) | 0 |  |  |  |  |  |  |  |
| XEYSKLandRD | 0.82 (0.66, 1.04) | 0.82 (0.65, 1.05) | 0.82 (0.69, 0.97) | 0.84 (0.73, 0.98) | 0 |  |  |  |  |  |  |
| XPYEKL | 0.98 (0.82, 1.20) | 0.98 (0.81, 1.21) | 0.98 (0.89, 1.08) | 1.01 (0.89, 1.14) | 1.20 (1.03, 1.40) | 0 |  |  |  |  |  |
| XPYEKLandRD | 0.96 (0.80, 1.17) | 0.96 (0.79, 1.18) | 0.96 (0.87, 1.05) | 0.99 (0.87, 1.12) | 1.17 (1.00, 1.36) | 0.98 (0.91, 1.04) | 0 |  |  |  |  |
| XSYWW | 0.96 (0.71, 1.28) | 0.96 (0.71, 1.28) | 0.96 (0.74, 1.20) | 0.99 (0.75, 1.25) | 1.17 (0.88, 1.51) | 0.98 (0.76, 1.21) | 1.00 (0.78, 1.24) | 0 |  |  |  |
| YSKKL | 1.14 (0.90, 1.44) | 1.14 (0.90, 1.45) | 1.13 (0.96, 1.34) | 1.16 (0.97, 1.40) | 1.38 (1.12, 1.70) | 1.16 (0.99, 1.35) | 1.18 (1.01, 1.38) | 1.18 (0.91, 1.58) | 0 |  |  |
| ZELKFY | 1.00 (0.75, 1.34) | 1.00 (0.75, 1.35) | 1.00 (0.78, 1.26) | 1.03 (0.80, 1.31) | 1.22 (0.93, 1.58) | 1.02 (0.80, 1.27) | 1.05 (0.82, 1.30) | 1.04 (0.76, 1.44) | 0.88 (0.67, 1.15) | 0 |  |
| RD | 1.16 (0.98, 1.41) | 1.16 (0.97, 1.42) | 1.16 (1.07, 1.26) | 1.19 (1.07, 1.33) | 1.41 (1.22, 1.64) | 1.18 (1.13, 1.24) | 1.21 (1.15, 1.27) | 1.21 (0.98, 1.55) | 1.02 (0.88, 1.19) | 1.16 (0.93, 1.46) | 0 |

**Table S8** Results of meta-regression for the overall effective rate of various commercial Chinese polyherbal preparations

| Outcome Measure | Interventions | Course(RR(95%CI)) |
| --- | --- | --- |
| Overall Effective Rate | JWXSKFY | -0.72(-68.34,57.83) |
|  | AMLKFY | 2.03(-1.76,5.88) |
|  | BEAKL | -3.15(-15.49,9.96) |
|  | BLJPKL | -8.21(-14.82,-2.71) |
|  | BZYQW | -1.23(-51.96,38.79) |
|  | EBKL | 1.04(-30.23,33.62) |
|  | EBKLandRD | -0.21(-31.09,30.91) |
|  | EKN | 0.62(-11.11,15.95) |
|  | EPXKL | 0.87(-48.61,61.57) |
|  | FFTZSKL | -0.94(-23.98,14.47) |
|  | HJKFY | 0.15(-43.59,46.13) |
|  | HQJKFY | 0.13(-3.73,4.07) |
|  | HWLGKL | 2.14(-20.59,41.66) |
|  | JBLKL | 0.55(-1.37,2.47) |
|  | JJKWKL | -0.82(-31.29,24.29) |
|  | JPW | 8.55(-16.45,73.43) |
|  | JPWandRD | -1.11(-16.72,15.02) |
|  | JWXSKFYandRD | 1.71(-58.78,73.87) |
|  | KELKL | -2.20(-49.13,21.72) |
|  | PKX | -4.26(-55.72,33.99) |
|  | QHZKL | 2.84(-24.19,42.81) |
|  | QPKFY | 1.62(-27.83,30.72) |
|  | QPWandRD | -0.30(-4.59,3.86) |
|  | QZKFY | 6.42(-6.55,26.68) |
|  | SBXSHJandRD | -11.80(-20.07,-3.52) |
|  | SJGRJ | -1.78(-6.46,1.82) |
|  | SLBZKLandRD | -0.18(-4.34,4.32) |
|  | SMJPKFY | -0.99(-77.48,74.22) |
|  | SQXSKFY | -1.23(-6.01,2.92) |
|  | SQXSKFYandRD | -1.59(-5.93,2.18) |
|  | WSBCW | -0.06(-2.01,2.11) |
|  | WSBCWandRD | 2.46(-15.43,35.94) |
|  | XECWKKL | 29.70(-21.82,218.61) |
|  | XEFFJNJJJP | 4.67(-19.23,57.61) |
|  | XEFFJNJJJPandRD | -1.92(-53.57,35.48) |
|  | XEFPKLandRD | -7.13(-72.96,45.08) |
|  | XEJPKWHJandRD | -1.30(-55.37,44.05) |
|  | XEJPW | -1.24(-7.72,4.62) |
|  | XEJWXSKFYandRD | -0.44(-32.07,21.92) |
|  | XEKKL | 3.03(-0.02,6.26) |
|  | XEKKLandRD | 1.90(-56.28,59.55) |
|  | XEKWZSKL | 0.68(-19.37,28.12) |
|  | XEPWLKL | 2.30(-46.36,55.75) |
|  | XEQZKFY | -0.20(-58.18,52.41) |
|  | XEWBW | -3.07(-34.99,18.06) |
|  | XEXISKL | 1.86(-1.49,5.51) |
|  | XEXJWandRD | -2.82(-60.41,52.76) |
|  | XEXSKL | 4.72(-55.66,134.52) |
|  | XEXSKLandRD | 2.62(-13.30,26.45) |
|  | XEYSKL | -1.47(-2.95,-0.05) |
|  | XEYSKLandRD | -1.28(-4.00,1.12) |
|  | XPYEKL | -0.14(-0.94,0.64) |
|  | XPYEKLandRD | 0.25(-0.55,1.10) |
|  | YSKKL | 4.37(-16.90,76.29) |

**Table S9** League table for commercial Chinese polyherbal preparations for weight changes in the treatment of infantile anorexia

| Interventions | RR(95%CI) | | | | | | | |
| --- | --- | --- | --- | --- | --- | --- | --- | --- |
|  | EBKLandRD | EPXKL | JBLKL | SQXSKFYandRD | WSBCWandRD | XEFFJNJJJP | XEFFJNJJJPandRD | RD |
| EBKLandRD | 0 |  |  |  |  |  |  |  |
| EPXKL | -2.15 (-5.68, 1.35) | 0 |  |  |  |  |  |  |
| JBLKL | -1.69 (-7.69, 4.27) | 0.46 (-5.16, 6.07) | 0 |  |  |  |  |  |
| SQXSKFYandRD | -0.93 (-3.94, 2.04) | 1.21 (-0.97, 3.41) | 0.74 (-4.55, 6.06) | 0 |  |  |  |  |
| WSBCWandRD | -1.6 (-5.22, 2.06) | 0.56 (-2.45, 3.58) | 0.09 (-5.59, 5.83) | -0.66 (-3.02, 1.74) | 0 |  |  |  |
| XEFFJNJJJP | 0.23 (-3.38, 3.8) | 2.38 (-0.57, 5.35) | 1.91 (-3.73, 7.58) | 1.16 (-1.13, 3.47) | 1.82 (-1.28, 4.9) | 0 |  |  |
| XEFFJNJJJPandRD | -2 (-5.21, 1.18) | 0.16 (-2.29, 2.61) | -0.32 (-5.72, 5.13) | -1.06 (-2.69, 0.58) | -0.4 (-3.04, 2.2) | -2.22 (-4.77, 0.33) | 0 |  |
| RD | 0.11 (-2.78, 2.96) | 2.26 (0.23, 4.29) | 1.78 (-3.43, 7.03) | 1.04 (0.21, 1.9) | 1.7 (-0.54, 3.92) | -0.12 (-2.27, 2.02) | 2.1 (0.71, 3.51) | 0 |

**Table S10** League table for commercial Chinese polyherbal preparations for weight changes in the treatment of infantile anorexia

| Interventions | RR(95%CI) | | | | | | | | |
| --- | --- | --- | --- | --- | --- | --- | --- | --- | --- |
|  | XEPWLKL | XEQZKFY | XEXJWandRD | XEXSKLandRD | XEYSKL | XEYSKLandRD | XPYEKL | XPYEKLandRD | RD |
| XEPWLKL | 0 |  |  |  |  |  |  |  |  |
| XEQZKFY | 1.2 (-1.55, 3.97) | 0 |  |  |  |  |  |  |  |
| XEXJWandRD | 0.01 (-2.66, 2.69) | -1.19 (-4.15, 1.78) | 0 |  |  |  |  |  |  |
| XEXSKLandRD | 0.54 (-1.62, 2.73) | -0.66 (-3.18, 1.86) | 0.53 (-1.93, 2.96) | 0 |  |  |  |  |  |
| XEYSKL | 0.92 (-0.95, 2.79) | -0.28 (-2.57, 1.98) | 0.92 (-1.3, 3.07) | 0.39 (-1.2, 1.91) | 0 |  |  |  |  |
| XEYSKLandRD | 0.07 (-1.84, 2) | -1.13 (-3.46, 1.18) | 0.06 (-2.17, 2.28) | -0.46 (-2.08, 1.13) | -0.86 (-1.82, 0.17) | 0 |  |  |  |
| XPYEKL | 0.15 (-1.91, 2.26) | -1.05 (-3.49, 1.42) | 0.14 (-2.23, 2.53) | -0.39 (-2.16, 1.44) | -0.78 (-2.13, 0.7) | 0.08 (-1.35, 1.58) | 0 |  |  |
| XPYEKLandRD | 0.05 (-1.84, 1.9) | -1.15 (-3.47, 1.09) | 0.04 (-2.19, 2.17) | -0.48 (-2.09, 0.99) | -0.87 (-1.96, 0.15) | -0.01 (-1.22, 1.06) | -0.09 (-1.59, 1.22) | 0 |  |
| RD | 1.23 (-0.47, 2.96) | 0.03 (-2.12, 2.19) | 1.23 (-0.84, 3.27) | 0.7 (-0.65, 2.04) | 0.3 (-0.42, 1.1) | 1.16 (0.3, 2.03) | 1.08 (-0.13, 2.25) | 1.17 (0.51, 1.99) | 0 |

**Table S11** Results of meta-regression for commercial Chinese polyherbal preparations for changes in weight

| Outcome Measure | Interventions | Course(RR(95%CI)) |
| --- | --- | --- |
| Changes in Weight | EBKLandRD | 6.12(-19.23,137.23) |
|  | EPXKL | -0.36(-14.61,12.71) |
|  | JBLKL | 0.37(-19.80,22.07) |
|  | SQXSKFYandRD | 1.97(-12.98,43.11) |
|  | WSBCWandRD | -0.54(-26.80,20.80) |
|  | XEFFJNJJJP | -0.82(-23.76,15.19) |
|  | XEFFJNJJJPandRD | -0.05(-14.18,13.54) |
|  | XEPWLKL | -0.17(-20.11,20.24) |
|  | XEQZKFY | 0.43(-15.37,18.80) |
|  | XEXJWandRD | 3.09(-18.19,55.55) |
|  | XEXSKLandRD | 7.82(-23.84,118.04) |
|  | XEYSKL | -0.90(-2.53,0.93) |
|  | XEYSKLandRD | 0.60(-1.16,2.31) |
|  | XPYEKL | -4.51(-20.81,4.00) |
|  | XPYEKLandRD | -0.30(-1.30,0.57) |

**Table S12** League table for commercial Chinese polyherbal preparations for improving hemoglobin in the treatment of infantile anorexia

| Interventions | RR(95%CI) | | | | | | |
| --- | --- | --- | --- | --- | --- | --- | --- |
|  | AMLKFY | EBKL | EBKLandRD | JBLKL | JJKWKL | JWXSKFYandRD | RD |
| AMLKFY | 0 |  |  |  |  |  |  |
| EBKL | 11.69 (-2.38, 25.72) | 0 |  |  |  |  |  |
| EBKLandRD | -13.26 (-25.65, -1.75) | -24.98 (-37.84, -12.83) | 0 |  |  |  |  |
| JBLKL | -28.73 (-44.43, -13.16) | -40.42 (-56.58, -24.3) | -15.45 (-29.47, -0.8) | 0 |  |  |  |
| JJKWKL | 1.41 (-11.86, 14.91) | -10.27 (-24.15, 3.73) | 14.67 (3.41, 27) | 30.16 (14.74, 45.69) | 0 |  |  |
| JWXSKFYandRD | -3.09 (-16.81, 10.69) | -14.76 (-29.13, -0.49) | 10.19 (-1.65, 22.93) | 25.66 (9.83, 41.63) | -4.5 (-18.19, 9.18) | 0 |  |
| RD | 5.22 (-4.28, 14.75) | -6.48 (-16.79, 3.8) | 18.5 (11.78, 26.03) | 33.97 (21.55, 46.4) | 3.79 (-5.6, 13.13) | 8.3 (-1.66, 18.28) | 0 |

**Table S13** League table for commercial Chinese polyherbal preparations for improving hemoglobin in the treatment of infantile anorexia

| Interventions | RR(95%CI) | | | | | | | |
| --- | --- | --- | --- | --- | --- | --- | --- | --- |
|  | SQXSKFYandRD | WSBCW | XEJPKWHJandRD | XEPWLKL | XEQZKFY | XEXSKLandRD | XPYEKLandRD | RD |
| SQXSKFYandRD | 0 |  |  |  |  |  |  |  |
| WSBCW | 11.69 (-2.38, 25.72) | 0 |  |  |  |  |  |  |
| XEJPKWHJandRD | -13.26 (-25.65, -1.75) | -24.98 (-37.84, -12.83) | 0 |  |  |  |  |  |
| XEPWLKL | -28.73 (-44.43, -13.16) | -40.42 (-56.58, -24.3) | -15.45 (-29.47, -0.8) | 0 |  |  |  |  |
| XEQZKFY | 1.41 (-11.86, 14.91) | -10.27 (-24.15, 3.73) | 14.67 (3.41, 27) | 30.16 (14.74, 45.69) | 0 |  |  |  |
| XEXSKLandRD | -3.09 (-16.81, 10.69) | -14.76 (-29.13, -0.49) | 10.19 (-1.65, 22.93) | 25.66 (9.83, 41.63) | -4.5 (-18.19, 9.18) | 0 |  |  |
| XPYEKLandRD | 5.22 (-4.28, 14.75) | -6.48 (-16.79, 3.8) | 18.5 (11.78, 26.03) | 33.97 (21.55, 46.4) | 3.79 (-5.6, 13.13) | 8.3 (-1.66, 18.28) | 0 |  |
| RD | 10.72 (4.8, 16.02) | 10.71 (0.13, 21.2) | 9.87 (1.92, 17.87) | 3.21 (-4.9, 10.99) | 1.11 (-8.18, 10.46) | 4.06 (-5.3, 13.42) | 7.12 (1.17, 12.94) | 0 |

**Table S14** Results of meta-regression for commercial Chinese polyherbal preparations for improving the levels of hemoglobin

| Outcome Measure | Interventions | Course(RR(95%CI)) |
| --- | --- | --- |
| Levels of Hb | AMLKFY | -0.45(-304.70,315.88) |
|  | EBKL | -4.56(-326.33,271.92) |
|  | EBKLandRD | -6.65(-255.07,213.22) |
|  | JBLKL | 5.68(-232.06,287.19) |
|  | JJKWKL | -39.61(-542.37,160.81) |
|  | JWXSKFYandRD | -9.51(-152.02,86.88) |
|  | SQXSKFYandRD | -12.17(-379.84,237.85) |
|  | WSBCW | -0.75(-148.09,133.89) |
|  | XEJPKWHJandRD | -13.29(-342.00,287.13) |
|  | XEPWLKL | 0.14(-251.81,263.62) |
|  | XEQZKFY | -8.15(-343.28,211.91) |
|  | XEXSKLandRD | 15.70(-222.43,408.43) |
|  | XPYEKLandRD | 1.86(-12.07,15.43) |

**Table S15** Results of meta-regression for adverse reactions of commercial Chinese polyherbal preparations

| Outcome Measure | Interventions | Course(RR(95%CI)) |
| --- | --- | --- |
| Adverse Reactions | HJKFY | -0.87(-36.98,34.32) |
|  | EBKLandRD | -2.88(-41.90,20.11) |
|  | EPXKL | 0.88(-19.41,32.42) |
|  | FFTZSKL | 2.63(-14.81,35.39) |
|  | HQJKFY | -0.47(-33.00,30.38) |
|  | JBLKL | -1.92(-34.16,20.52) |
|  | JJKWKL | -0.31(-15.60,12.73) |
|  | QPKFY | -1.42(-30.69,20.96) |
|  | QZKFY | 6.03(-24.20,61.80) |
|  | SJGRJ | 0.39(-32.04,33.05) |
|  | SQXSKFYandRD | 0.85(-20.53,28.72) |
|  | WSBCWandRD | -2.81(-36.62,13.32) |
|  | XEFFJNJJJP | 1.49(-44.51,77.12) |
|  | XEFFJNJJJPandRD | -3.71(-45.97,20.33) |
|  | XEFPKLandRD | -0.87(-20.76,13.15) |
|  | XEJPKWHJandRD | -5.92(-48.61,28.75) |
|  | XEKKL | 8.82(-21.58,74.26) |
|  | XEPWLKL | 0.66(-22.33,27.38) |
|  | XEQZKFY | 1.49(-29.81,30.12) |
|  | XEXSKLandRD | 2.00(-26.38,34.88) |
|  | XEYSKL | -1.40(-31.50,21.17) |
|  | XEYSKLandRD | -2.23(-34.46,21.35) |
|  | XPYEKL | -0.65(-24.15,19.92) |
|  | XPYEKLandRD | -0.96(-18.01,10.54) |
|  | YSKKL | 1.48(-22.69,31.71) |
